# Aflatoxins: Occurrence, Exposure, and Binding to *Lactobacillus* Species from the Gut Microbiota of Rural Ugandan Children

**DOI:** 10.3390/microorganisms8030347

**Published:** 2020-02-29

**Authors:** Alex Paul Wacoo, Prudence Atukunda, Grace Muhoozi, Martin Braster, Marijke Wagner, Tim J van den Broek, Wilbert Sybesma, Ane C. Westerberg, Per Ole Iversen, Remco Kort

**Affiliations:** 1 Department of Molecular Cell Biology, Vrije Universiteit Amsterdam, 1081 HZ Amsterdam, The Netherlands; wacooalex@gmail.com (A.P.W.); m.braster@vu.nl (M.B.); m.j.wagner@vu.nl (M.W.); 2 Yoba for Life foundation, 1079 WB Amsterdam, The Netherlands; wilbert.sybesma@yoba4life.org; 3 Department of Medical Biochemistry, School of Biomedical Sciences, College of Health Sciences, Makerere University, P.O. Box 7062 Kampala, Uganda; 4 Department of Nutrition, Institute of Basic Medical Sciences, University of Oslo, 0317 Oslo, Norway; prudence.atukunda@studmed.uio.no (P.A.); p.o.iversen@medisin.uio.no (P.O.I.); 5 Department of Human Nutrition and Home Economics, Kyambogo University, P.O. Box 1 Kampala, Uganda; g.k.m.muhoozi@medisin.uio.no; 6 Department of Microbiology and Systems Biology, TNO, 3704 HE Zeist, The Netherlands; tim.vandenbroek@tno.nl; 7 Institute of Health Sciences, Kristiania University College, 0107 Oslo, Norway; AneCecilie.Westerberg@kristiania.no; 8 Division of Human Nutrition, Stellenbosch University, Tygerberg, 7505 Cape Town, South Africa; 9 Department of Hematology, Oslo University Hospital, 0318 Oslo, Norway; 10 ARTIS-Micropia, 1018 CZ Amsterdam, The Netherlands

**Keywords:** Stunting, aflatoxin B_1_, Lactic acid bacteria, aflatoxin binding, gut microbiota

## Abstract

Chronic exposure of children in sub-Saharan Africa to aflatoxins has been associated with low birth weight, stunted growth, immune suppression, and liver function damage. *Lactobacillus* species have been shown to reduce aflatoxin contamination during the process of food fermentation. Twenty-three *Lactobacillu*s strains were isolated from fecal samples obtained from a cohort of rural Ugandan children at the age of 54 to 60 months, typed by 16S rRNA gene sequencing, and characterized in terms of their ability to bind aflatoxin B_1_ in vitro. Evidence for chronic exposure of these children to aflatoxin B_1_ in the study area was obtained by analysis of local foods (maize flour and peanuts), followed by the identification of the breakdown product aflatoxin M_1_ in their urine samples. Surprisingly, *Lactobacillus* in the gut microbiota of 140 children from the same cohort at 24 and 36 months showed the highest positive correlation coefficient with stunting among all bacterial genera identified in the stool samples. This correlation was interpreted to be associated with dietary changes from breastfeeding to plant-based solid foods that pose an additional risk for aflatoxin contamination, on one hand, and lead to increased intake of *Lactobacillus* species on the other.

## 1. Introduction

The warm and humid climate conditions of sub-Saharan Africa promote the growth of fungi and associated production of mycotoxins. Approximately 25% of grains harvested annually worldwide contain mycotoxins. Ingestion of these contaminated foods can lead to disease and death [1]. Aflatoxin is the most prevalent and harmful human mycotoxin reported to date [2]. Aflatoxins are common food contaminants produced as secondary metabolites of fungi belonging to genus *Aspergillus* [3]. Their toxicity leads to carcinogenic and teratogenic effects as well as growth faltering, which has been confirmed in animal models, rendering aflatoxins a major food safety concern [4,5,6].

Four major types of aflatoxins such as B_1_, B_2_, G_1_, and G_2_ are commonly reported as contaminants of foods, including maize, ground nut, and cotton seeds. Aflatoxin B_1_ is the most prevalent, contributing to up to 75% of all aflatoxin contamination of foods, and it has been classified as a Group 1 carcinogen by the International Agency for Research on Cancer (IARC) in 1987 [3]. To minimize the risk of aflatoxin ingestion from contaminated foods, maximum levels for aflatoxin in nuts, grains, and oil seeds has been set up by many countries. The East Africa Community has proposed the maximum allowable level of aflatoxin as 5 µg kg^−1^ for aflatoxin B_1_ and 10 µg kg^−1^ for total aflatoxin [7]. Some African countries adopted codex regulatory levels, which vary between 0.5 and 15 µg kg^−1^ [8]. In comparison, the European Commission set the regulatory limit at 2 for B_1_ and 4 µg kg^−1^ for total aflatoxins in human foods [9].

Despite all these regulatory limits, aflatoxins are still present in dangerously high levels in groundnuts, cassava, and corn, which make up the bulk of children’s diets in Africa [10]. Approximately 74% of maize flour consumed in Kampala, Uganda, was contaminated with aflatoxins at a range from 1.8 to 268 µg kg^−1^ [11]. These high levels of contamination were further confirmed by Muzoora et al. who found that 72% of peanuts collected from different regions of Uganda were contaminated with aflatoxins, ranging from 1.6 to 516 µg kg^−1^ [12]. Due to ingestion of highly contaminated foods, Asiki et al. reported that all 100 adults and 92 children out of a total of 96 tested children had detectable levels of aflatoxin-albumin adduct [13]. This study also revealed that five babies who were exclusively breastfed tested positive for aflatoxin albumin adduct. Although a direct causal relationship has not been established, high levels of aflatoxin exposure could contribute to the high rate of stunted growth of 46% in Western Uganda [14,15].

Following ingestion of contaminated food, and reaching the upper small intestine (duodenum), aflatoxin is absorbed into the blood stream rapidly [16]. Although there is rapid absorption, aflatoxins have been found to affect the gastrointestinal tract by impairing cell growth, causing DNA damage and increasing lactate dehydrogenase activity [17]. Moreover, aflatoxins have also been reported to affect the gut microbiota. Wang et al. showed that aflatoxin B_1_ has the ability to alter the gut microbiota in a dose-dependent manner in rats; aflatoxin B_1_ did not affect gut microbiota at the phylum level, but some lactic acid bacteria were depleted [18]. Galarza-Seeber et al. also revealed that aflatoxins at a dose of one part per million (ppm) significantly decreased total lactic acid bacteria in broilers [19].

The human gut microbiota is composed of trillions of bacteria that play an important role in maintaining health [20]. The gut microbiota provides a protective barrier for the host against the proliferation of pathogenic bacteria. Gut bacteria also play a crucial role in the digestion of a wide range of foods as well as the binding and degradation of toxins [21]. Therefore, the intake of probiotics and lactic acid bacteria via fermented foods could help to reduce the uptake of aflatoxins [22]. In a study carried out on young men from Guangzhou, China, significant reduction of urinary aflatoxin was noted after administration of *Lactobacillus rhamnosus* LC705 and *Propionibacterium freudenreichii* compared to placebo [23]. A similar study showed that administration of *L. casei* Shirota significantly decreased the level of aflatoxin B_1_-lysine adduct [24]. Furthermore, a number of other studies confirmed the ability of lactic acid bacteria to bind aflatoxin B_1_ [25,26].

Despite the evidence for the toxic effects of aflatoxins, there is generally poor awareness of the risk of these toxins and a lack of proper detection methods to monitor levels in food [27]. Therefore, a portable immunosensor was developed, validated, and used to measure aflatoxin levels in maize from markets and households in Kampala [11,28], indicating that consumers, including children, are exposed to relatively high concentrations of aflatoxin. A strategy was proposed to detoxify aflatoxin in end products by fermentation with the probiotic gut isolate *Lactobacillus rhamnosus* yoba 2012 [29]. In this study, the exposure to aflatoxins was evaluated for 10 children (aged 54–60 months) from a cohort of 511 children. Their foods (maize flour and peanuts) were analyzed for the presence of aflatoxin B_1_ and their urine for aflatoxin M_1_. The aflatoxin B_1_ binding ability was tested of *Lactobacillus* species isolated from the gut microbiota of these children, and the correlation of gut *Lactobacillus* species with stunting for 140 children was analyzed from the same cohort at the ages of 20–24 and at 36 months.

## 2. Materials and Methods

### 2.1. Study Design and Sample Collection

**Figure 1:**
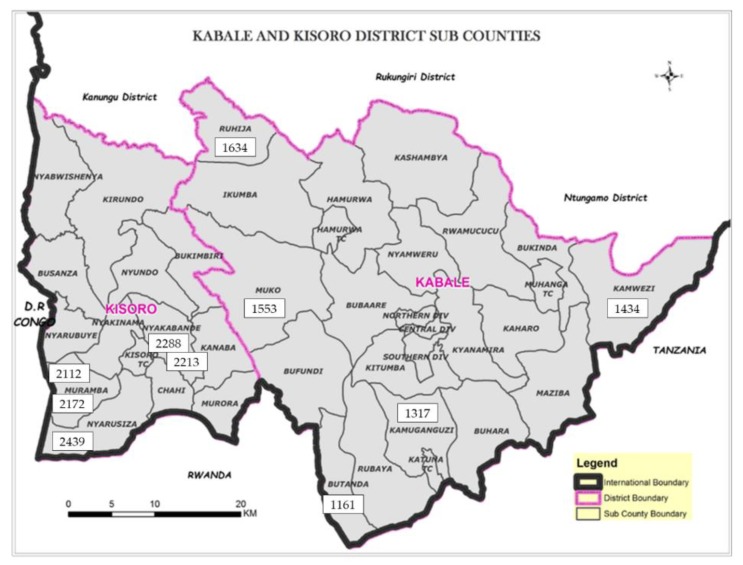
Map of Kabale and Kisoro showing subcounties where stool, urine, hulled maize, dehulled maize flour, and peanuts were sampled in this study. The geographic locations of the ten children (aged 54 to 60 months) are indicated by child identification number.

In the current study, a small sample of ten children aged 54–60 months was selected on the basis of their previous growth indices at 36 months (5 stunted and 5 non-stunted children). These children took part in a randomized trial in the two districts of Kabale and Kisoro, located in the southwestern part of Uganda (Figure 1). The trial assessed the effect of an educational intervention (focusing on nutrition, hygiene, and stimulation) on their growth and development as described in detail in previous publications [30,31]. Samples of the stool and urine were taken from every child in this study. The stool was sampled using a sterile disposable stool sampling container. The stool samples from every child were put in two separate containers: one container was filled with mineral oil and kept at room temperature for the purpose of cultivation, and the other container was filled with two milliliters of 15% glycerol. The stool samples with glycerol were immediately kept on ice and transferred to the Uganda Industrial Research Institute for storage at −80 °C. Urine was sampled into a sterile, disposable plastic container and immediately stored at low temperature (0–8 °C). The ten children were widely spread across the districts of Kabale and Kisoro as indicated by subject identification number (Figure 1).

Anthropometric measurements were taken as described by Muhoozi et al. [30] and used to compute height-for-age Z–scores (HAZ) [32]. The frequently consumed regional foods (hulled and dehulled maize, and peanuts) at risk of aflatoxin contamination were identified based on a short food frequency questionnaire (Supplementary File S1). Common diet for the age group and last week’s diet of the children were evaluated. The foods were sampled and immediately stored at low temperature (0–8 °C). The in vitro measurements were carried out at the Uganda Industrial Research Institute (Kampala, Uganda) and at the Department of Molecular Cell Biology, Vrije Universiteit (Amsterdam, The Netherlands). The *Lactobacillus* species were isolated, and their ability to bind aflatoxin B_1_ was assessed. The concentration of aflatoxin B_1_ was determined in the food samples and aflatoxin M_1_ in the children’s urine.

### 2.2. Aflatoxin B_1_ in Food and Daily Intake

Hulled, dehulled maize flour, and peanut were analyzed using the ELISA Ridascreen^®^ Aflatoxin B_1_ with a reported limit of detection of 1.0 µg kg^−1^ (R-Biopharm, Darmstadt, Germany). The enzyme immunoassay was first calibrated by the use of an aflatoxin B_1_ standard (0, 1, 5, 10, 20, and 50 µg L^−1^) in 10% (*v/v*) methanol as indicated in Supplementary File S2. A competitive enzyme immunoassay was used for the determination of aflatoxin B_1_ in cereals similar to the method described by Wacoo et al. [11]. Briefly, 5 g of homogenized maize flour was weighed into 50 mL centrifuge tubes, followed by addition of 25 mL of 70% (*v/v*) methanol and thorough mixing using a VWR ADC 3500 Shaker (BioSurplus, Inc, San Diego, CA, USA) for 5 min. In the case of peanuts, 0.4 g of sodium chloride was added to the suspension and thoroughly mixed. The suspension was then centrifuged for 10 min at 3500× *g* at room temperature. An aliquot of 50 µL of each supernatant was used for aflatoxin B_1_ determination using the ELISA kit.

The minimum daily aflatoxin B_1_ intake of children in southwestern Uganda (expressed in ng kg^−1^ day^−1^) was estimated on basis of the measured concentrations of aflatoxin B_1_ in maize flour and peanut sampled in each subcounty, the estimated amounts of maize and peanut consumed, and the measured body weight of the child [33]. The frequency of intake of maize flour and peanut in southwestern Uganda was obtained by a food frequency questionnaire (Supplementary File S1). The amount of maize and peanut consumed was based on the previously reported intake estimate for maize flour and products of 60 g per day for children at the age of 24 to 59 months in southwestern Uganda [34].

### 2.3. Aflatoxin M_1_ in Urine

Quantitative determination of aflatoxin M_1_ in urine samples was carried out using the ELISA Ridascreen^®^ Aflatoxin M_1_ designed with a limit of detection of 5 ng L^−1^. The ELISA kit was validated as described by the International Conference on Harmonization (ICH) (1995) [35]. The limit of detection (LOD), precision, and accuracy were determined by using concentrations of aflatoxin M_1_ standard (0, 125, 250, 500, 1000, and 2000 ppt) spiked in urine (Supplementary File S2). Briefly, the pH of the urine samples was first adjusted to seven. A volume of 50 µL of either standard or urine samples was pipetted and put into separate wells, followed by addition of 50 µL of enzyme conjugate and 50 µL of anti-aflatoxin M_1_ antibody solution. The microwell plate was then mixed by shaking gently and incubated for 10 min at room temperature. The liquid was removed and the wells were washed three times using 250 µL of washing buffer. Then, the wells were filled with 100 µL of substrate and incubated for 5 min at room temperature. The reaction was stopped with 100 µL of stop solution and the absorbance taken at 450 nm wavelength using a microplate reader. The results of the standard solution were used to develop a calibration curve, and the aflatoxin M_1_ levels in each urine sample were determined from this curve. Urine creatinine concentrations were determined spectrophotometrically by the modified Jaffe method [36]. The aflatoxin M_1_ concentration from each sample was subsequently normalized to creatinine concentration in the urine. The percentage of aflatoxin, which is excreted as aflatoxin M_1_ in urine, was calculated on basis on the estimated dietary aflatoxin intake per kg body weight per day (ng), the concentration of aflatoxin M_1_ in urine (ng/mg creatinine), and the reported levels of excreted creatinine in urine per day of 15.4 mg per kg body weight per day for boys and 14.3 mg per kg body weight per day for girls [37,38].

### 2.4. Enumeration and Isolation of Lactic Acid Bacteria

Serial dilutions of samples were prepared in physiological saline. Total counts of lactic acid bacteria (LAB) from stool samples were determined by streaking selected serial dilutions on sterile de Man, Rogosa, Sharpe (MRS) agar (Oxoid limited, Hampshire, United Kingdom) containing 0.1% Tween 80. The plates were incubated at 37 °C for 48 h. The experiment was performed four times for every sample. After total counts of lactic acid bacteria (LAB), five colonies with distinct colony morphology were selected from each plate. The colonies were then streaked to freshly prepared MRS agar plates for identification.

### 2.5. Identification of Bacterial Isolates

Isolates were identified as described by Felske et al. [39]. Briefly, 16S rRNA gene fragments were amplified and sequenced using primers 8F (5′-AGAGTTTGATYMTGGCTCAG-3′) and 1512R (5′-ACGGYTACCTTGTTACGACTT-3′). The colony PCR reactions were carried out with 1 μL of each primer (10 pmol), 11 μL nuclease-free water (Promega), and 12 μL GoTaq Colorless Master Mix (Promega) in a final volume of 24 μL. To the PCR reaction mix, a small amount of a fresh colony was added using a sterile toothpick. The PCR program was set as follows: initial denaturation was carried out at 94 °C for 5 min, followed by 30 amplification cycles (30 s at 94 °C, 30 s at 55 °C, and 30 s at 72 °C), and a final extension step at 72 °C for 8 min. The PCR products were verified by electrophoresis on 1.5% (*w/v*) agarose gel, and sequencing was done by the Sanger sequencing method (Macrogen Inc., The Netherlands). Sequences were compared to sequences deposited in GenBank by using the (Basic Local Alignment Search Tool) BLAST algorithm, National Library of Medicine, Bethesda MD, USA [40]. All isolated strains are accessible from the strain collection of the Department of Molecular Cell Biology, Vrije Universiteit Amsterdam, The Netherlands, as well as the Department of Microbiology, School of Biomedical Sciences, College of Health Sciences, Makerere University, Uganda, in order to assure benefit sharing in accordance with the Nagoya protocol [41]. The correlation between the *Lactobacillus* species isolated at 54–60 months and microbiota at 20–24 and 36 months was assessed by matching the V4 amplicon sequences of our nearly full-length 16S rRNA sequences with our previously collected microbiota data (Sequence Read Archive SUB4476421) [31].

### 2.6. Aflatoxin B_1_ Binding to the Isolated Lactobacillus Species

The aflatoxin B_1_ binding assay was performed as described by Wacoo et al. [29]. Briefly, the isolated *Lactobacillus* species were cultured in de Man, Rogosa, Sharpe (MRS) broth with 0.1% (v/v) Tween 80 at 37 °C for 24 h. The cells were pelleted at 3200 *g* for 10 min at room temperature and subsequently washed twice with physiological saline to remove excess MRS broth. The washed cell pellets were re-suspended in 2 mL of physiological saline solution. Each suspension was then serially diluted with physiological saline to obtain approximately 10^8^ cfu mL^−1^. These dilutions were centrifuged, and the cell pellets re-suspended in 1.0 mg mL^−1^ of aflatoxin B_1_ followed by incubation at 37 °C for 30 min. After incubation, the aflatoxin B_1_ cell suspensions were centrifuged at 3200× *g* for 10 min at room temperature, and the residual aflatoxin B_1_ in the supernatant was determined using the Fluostar Omega microplate reader (BMG Labtech, Ortenberg, Germany) at an excitation of 390 nm and an emission 480 nm. Bound aflatoxin B_1_ was calculated by use of the formula below:

(1) $$\frac{\left(\mathrm{Initial}\;\mathrm{AFB}1-\mathrm{Residual}\;\mathrm{AFB}1\right)\;\times \;100}{\mathrm{Initial}\;\mathrm{AFB}1\;}$$

### 2.7. Statistical Analysis

For comparison between *Lactobacillus* species at 20–24 and 36 months, species were identified with BLAST of 16S rRNA amplicon sequences, and the data were presented in a pie chart of *Lactobacillus* species as the average percentage of the total *Lactobacillus* per individual. The abundances of *Lactobacillus* species were calculated by the percentage of all V4 16S rRNA sequence reads from the 23 isolates showing a 100% identity match in the total pool of unique sequence reads at 20–24 months and 36 months. The prevalence of *Lactobacillus* species was calculated as the percentage of gut microbiota compositions from the 140 children containing at least one exact match to the specific V4 sequence read from one of the 23 *Lactobacillus* isolates. A permutational multivariate analysis of variance (PERMANOVA) was carried on gut microbiota composition of all rural Ugandan children (*n* = 140) at 20–24 and 36 months and growth development scores. Analysis was performed using R version 3.3.2, with PERMANOVA as implemented in the ‘vegan’ package by Oksanen et al. using the Bray–Curtis distance measure [42]. The 16S rRNA gene sequencing data were rescaled and transformed using Wisconsin double transformation and square root transformation. The correlations between *Lactobacillus* species abundance in stunted (HAZ < −2.0) and nonstunted (HAZ > −2.0) children were displayed in violin plots by the use of OriginPro 2019b 9.6.5.169 (Academic).

### 2.8. Ethical Clearance

The study was approved by the Research Ethics committee of The AIDS Support Organization (no. TASOREC/06/15-UG-REC-009) and by the Uganda National Council for Science and Technology (no. UNCST HS 1809).

## 3. Results

### 3.1. Aflatoxin B_1_ Contamination in Food

In our survey on the consumption of particular foods for southwestern Uganda, beans were found to be the most frequently consumed food (Table S1). This was followed by posho/porridge (corn bread or porridge), greens (eshiga), and Irish potatoes, which were consumed at least once a day. Sweet potatoes and fermented porridge were consumed five to six times a week. Peanut, millet and sorghum porridge were taken four times a month, and dry maize with beans, cassava, and rice was eaten two to three times a month. The foods with a very high risk of aflatoxin contamination were posho/porridge, which was consumed daily, and peanuts. The intake of these foods may result in accumulation of aflatoxin in the body; thus, the ingredients of posho/porridge (maize) and peanuts were selected for further analysis.

**Figure 2:**
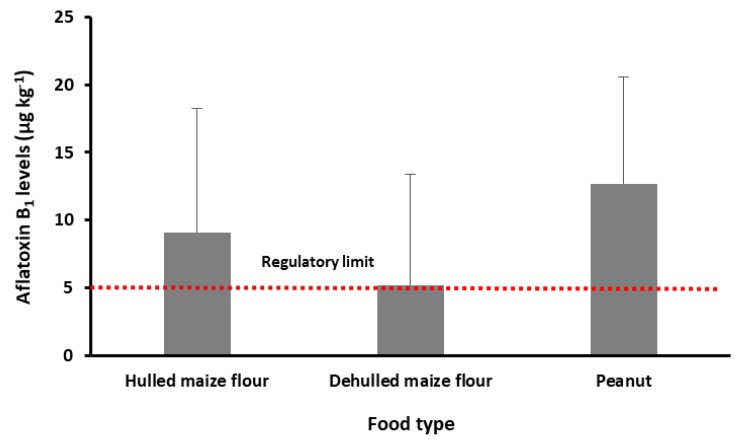
Aflatoxin B_1_ levels in maize flour and peanut samples from Kabale and Kisoro, southwestern Uganda, and the East African regulatory limit (red dotted line). Values are means ± standard deviation (*n* = 10).

The levels of aflatoxin B_1_ in hulled, dehulled maize, and peanut sampled from Kabale and Kisoro district of southwestern Uganda are shown in Figure 2. All of these food ingredients contained mean aflatoxin B_1_ levels above the acceptable East African regulatory limit of 5 µg kg^−1^ for aflatoxin B_1_ [7]. The mean aflatoxin B_1_ concentration in hulled maize flour was 9.1 µg kg^−1^. Only 10% of the hulled maize flour samples contained undetectable levels of aflatoxin B_1_. Dehulled maize flour contained comparable concentrations of aflatoxin B_1_ with a mean of 5.3 µg kg^−1^, 6% higher than the East African regulatory limit of 5 µg kg^−1^ [7]. Approximately 50% of dehulled maize flour contained detectable levels of aflatoxin B_1_. Peanut samples contained, on average, higher concentrations of aflatoxin B_1_ with 12.8 µg kg^−1^. Approximately, 90% of the peanut samples contained aflatoxin B_1_ with levels ranging from 1.8 to 20.2 µg kg^−1^. More than 50% of the peanut samples contained aflatoxin B_1_ levels higher than the East African regulatory limit of 5 µg kg^−1^.

### 3.2. Daily Intake of Aflatoxin B_1_

Estimates for the daily intake of aflatoxin B_1_ by the children of southwestern Uganda are shown in Table 1. The dietary aflatoxin B_1_ intake varied from 1.12 to 88.6 ng kg^−1^ day^−1^. The overall mean dietary aflatoxin B_1_ exposure was 50.1 ng kg^−1^ day^−1^. The mean dietary aflatoxin B_1_ exposures to stunted and nonstunted children were 53.7 and 46.5 ng kg^−1^ day^−1^, respectively. Although there was a notable difference in the mean dietary aflatoxin B_1_ exposure to the stunted and nonstunted children, statistically the difference was not significant (*p* > 0.5).

### 3.3. Aflatoxin M_1_ in Urine

The results of aflatoxin M_1_ analyzed from the urine samples of both nonstunted and stunted children are shown in Table 1, which also shows anthropometric parameters for the same children. The aflatoxin M_1_ levels in the urine samples varied from 14.8 to 168 pg mg^−1^ of creatinine. There was no significant difference between aflatoxin M_1_ levels in nonstunted children and stunted children (*p* > 0.05). A detectable amount of aflatoxin B_1_ in maize flour and peanut coincided with detectable levels of aflatoxin M_1_ in urine samples for all children from the different study regions. No positive correlation was observed between the minimum estimated daily intake of aflatoxin B_1_ and the levels of aflatoxin M_1_ found in urine samples.

### 3.4. Enumeration of Lactic Acid Bacteria

Lactic acid bacteria from the stool were counted and the results shown in Table 1. The lactic acid bacterial count in the stool samples varied from 8.9 × 10^6^ to 1.9 ×10^8^ cfu g^−1^. There was no significant difference between the bacterial counts between the nonstunted children (on average 7.0 × 10^7^ cfu g^−1^) and stunted children (on average 6.9 × 10^7^ cfu g^−1^). Approximately 10% of the samples contained lactic acid bacteria at the level of 10^6^ cfu g^−1^. More than 50% of the samples contained lactic acid bacteria at the level of 10^7^ cfu g^−1^ and 30% contained lactic acid bacterial concentrations of 10^8^ cfu g^−1^.

### 3.5. Isolation, Identification, and Aflatoxin B_1_ Binding Properties of Lactobacillus Species

In this study, 23 *Lactobacillus* strains were isolated from fecal samples of 10 children aged 54 to 60 months (Table 2). The identification of the strains was performed based on 16S rRNA gene sequencing [31]. As nearly full-length 16S rRNA sequencing was applied, only one unambiguous identification was found on the species level of either *L. casei* or *L. paracasei* with both a percentage identity of 96% (Supplementary File S3). Though the same species of *Lactobacillus* was repeatedly isolated among subjects, they did not demonstrate equal binding potential to aflatoxin B_1_. Strains of the species of *L. casei* were most frequently isolated, accounting for 30% of the total *Lactobacillus* isolates, but it was also the most prevalent appearing in over 50% of the subjects. Both *L. plantarum* and *L. brevis* accounted for 14% of the total isolates each. However, *L. plantarum* was isolated in approximately 40% and *L. brevis* was isolated from only 20% of the subjects.

All isolated *Lactobacillus* strains were assessed for their ability to bind aflatoxin B_1_ [30]. All *Lactobacillus* isolates demonstrated binding of aflatoxin B_1_ in physiological saline as shown in Table 2 at slightly variable levels. The best aflatoxin B_1_ binding was registered for *L. fermentum* APW1317 and *L. casei* APW2213C of 76.1% and 62.6% at 10^8^ cfu mL^−1^ cell concentration. It should be noted that no systematic differences in binding ability were observed between *Lactobacillus* species or subjects. The amount of aflatoxin bound to the bacteria increased with a rise in cell density from 4.0 × 10^7^ to 8.1 × 10^7^ cfu mL^−1^. However, some strains indicated higher aflatoxin B_1_ binding at relatively low cell densities. This is attributed to the ability of these bacteria to coagulate and form clumps at high cell densities resulting in a smaller cell surface area for binding.

The *Lactobacillus* species in the gut microbiota of the children at 20–24 and 36 months accounted for 2.2% and 3.4% of the total sequence reads, respectively. All the isolated *Lactobacillus* species at the age of 54–60 months were found to be present at 20–24 and at 36 months, except for *L. rhamnosus* and *L. pantheris*, which could not be detected at 20–24 months. Generally, the abundance of *Lactobacillus* species isolated at 54–60 months, which were present at 20–24 months, varied from 0 to 0.03%. The food-derived *L. fermentum* was the most abundant (0.03%) and the most prevalent isolate (22.9%) found amongst the 140 children at 20–24 months (Table 2).

### 3.6. Distribution of Lactobacillus Species in Stool Samples

**Figure 3:**
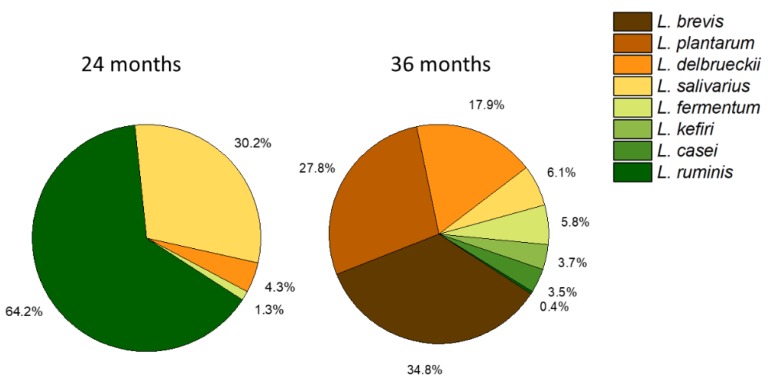
The average relative abundance of *Lactobacillus* sequences from children of Kabale and Kisoro, southwestern Uganda, at the age of 20–24 months and at 36 months. *Lactobacillus* species with a relative abundance of ≤ 0.01% were not included. The following combinations of species could not be unambiguously resolved as their 16S rRNA V4 amplicon sequence showed identical matches to the sequence in the 16S rRNA database: *L. delbrueckii* and *L. leichmannii, L. plantarum* and *L. pentosus, and L. casei* and *L. paracasei*.

The distribution of *Lactobacillus* species in the stool samples of the Ugandan children is shown in Figure 3. At the age of 20–24 months, *L. ruminis* was the most dominant species of *Lactobacillus,* accounting for approximately 64.2% of the total *Lactobacillus* species composition of the gut microbiota. *L. ruminis* had an abundance of 1.3% of the total *Lactobacillus* species at 20–24 months. *L. salivarius* accounted for approximately 30.2% with abundance of 0.6% at 20–24 months. *L. delbrueckii* and *L. fermentum* accounted for 4.3% and 1% with corresponding abundance of 0.09% and 0.03%, respectively. The relative abundance of *Lactobacillus* species to the gut microbiota increased from 2.17% to 3.42% of all 16S rRNA sequence counts in children from 20–24 months to 36 months. Approximately four more dominant species of *Lactobacillus* emerged at the age of 36 months at the expense of *L. salivarius* and *L. ruminis*. There was a notable shift from autochthonous (endogenous) to allochtonous (plant derived) *Lactobacillus* species, most probably resulting from the change in diet from breast milk to solid food. The allochtonous *Lactobacillus* species *L. brevis* emerged at the age of 36 months and became the most dominant species with 34.8%, while the endogenous species *L. salivarius* and *L. ruminis* dropped from 64.2% and 30.2% to 6.1% and 0.4%, respectively. *L. plantarum, L. delbrueckii,* and *L. fermentum* increased from less than 0.01%, 4.3%, and 1.3% at 20–24 months to 27.8%, 17.9%, and 5.8% at 36 months, respectively. The other prominent species *L. kefiri* and *L. casei* were also found at the age of 36 months, accounting for 3.7% and 3.5%, respectively.

### 3.7. Lactobacillus Inversely Correlates with Growth

**Figure 4:**
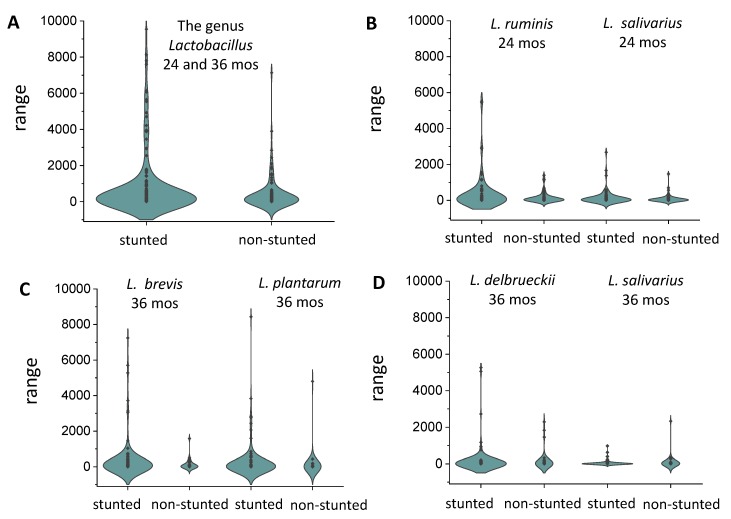
Violin plots showing the abundance of *Lactobacillus* genus and species in stunted and nonstunted children expressed in sequence reads in the gut microbiota of 140 children from Kabale and Kisoro, southwestern Uganda, at 20–24 months (*n* stunted = 77) and 36 months (*n* stunted = 104).

The permutational multivariate analysis of variance carried out on gut microbiota composition of rural Ugandan children (*n* = 140) with anthropometric and cognitive development scores indicated a number of significant correlations. The most pronounced variable was age (20–24 and 36 months) explaining 4.03% of the variance in the microbiota composition with *p* = 0.001 (Supplementary File S4). The anthropometric measures HAZ, and stunting, explained respectively 0.91% and 0.84% of the variance in the gut microbiota (*p* = 0.001). The genus *Lactobacillus* appeared as the genus with the highest correlation coefficient (0.014) for stunting among all 256 taxonomic units of bacterial genera identified in the gut microbiota. This was also evident from a representation of *Lactobacillus* abundance in the gut microbiota of Ugandan children at 20–24 and 36 months for stunted and nonstunted children (Figure 4A). The average number of *Lactobacillus* sequence reads was 801 for stunted children and 423 for nonstunted children. However, it should be noted that this difference was not significant (*p* > 0.05) in non-parametric tests for non-normally distributed data sets. A closer inspection on the *Lactobacillus* species level (Figure 4B–D) revealed that the most predominant *Lactobacillus* species followed this trend, except for *L. salivarius* at 36 months, which appeared more abundantly present in non-stunted children.

## 4. Discussion

A high exposure to aflatoxin-contaminated food negatively correlates with impaired growth in children [4]. The current study provides evidence that Ugandan rural children are exposed to high concentrations of aflatoxin B_1_ on a daily basis through consumption of contaminated food stuffs. Analysis of a set of regional maize flour samples indicated higher levels for hulled maize compared to dehulled maize. Siwela et al. were able to reduce up to 92% of aflatoxin contamination in maize through the process of dehulling [43]. Notably, the hulled maize with relatively high contamination levels is the most preferred due to the low costs. The dehulled maize is usually purchased by people categorized in the middle income class.

The estimated intake level to dietary aflatoxin in Uganda ranges from 10 to 180 ng per kg body weight per day [44]. In this study, the minimal estimated dietary aflatoxin intake levels for 80% of the children were found to be within this reported range for Uganda. The finding of 20% of the children with a minimal aflatoxin intake of less than 10 ng per kg body weight per day could result from the coincidental regional sampling of maize with a short storage time. However, lower intake values have been reported for other East African countries including Kenya (4−133 ng per kg body weight per day) [45].

The children’s exposure to aflatoxin B_1_ was confirmed by analysis of their urine samples for the presence of aflatoxin M_1_. As reported previously, approximately 1.2% to 2.2% of the dietary aflatoxin B_1_ intake can be excreted in urine as aflatoxin M_1_ [46]. If two minimal intake levels below 10 ng per kg body weight are considered outliers, the average percentage of aflatoxins excreted in the urine as aflatoxin M_1_ equals 3.3% ± 2.8%. As in this study minimal intake levels for aflatoxin for B_1_ were determined, this average percentage will decrease if aflatoxin intake from other food sources such as millet, sorghum, and beans will be included in the analysis.

The ability of lactic acid bacteria including *Lactobacillus* to protect against food mutagens such as aflatoxins, heterocyclic amines, and phytate among others has been reported in other studies [25,26,47,48]. Physical binding to the bacterial cell wall is reported as one of the mechanisms for the mitigation of aflatoxins from the intestine [49]. In the current study *Lactobacillus* species were isolated, characterized and their aflatoxin B_1_ binding ability was tested. The *Lactobacillus* strains demonstrated a variable ability to bind aflatoxin B_1._ This variation could be attributed to the differences in structure of the proposed binding surfaces such as cell wall polysaccharides, peptidoglycan, teichoic acid, and cell wall proteins, which are known to be variable among bacterial strains of the same species [48,50].

Previous studies demonstrated that gut microbiota of newborns evolves rapidly during the first 12 months of life, remains highly dynamic up to the age of 24 of months, and becomes more stable afterwards [51]. The diet contributes significantly to this modulation [52]. At the age of 20–24 months, most of the children in our cohort of 511 children were still taking breast milk, while at the age of 36 months this was replaced by solid foods [30]. This could explain the change in composition from the autochthonous species *L. salivarius* and *L. ruminis* at 20–24 months to the allochthonous species *L. plantarum*, *L. brevis, L. delbrueckii, L. casei,* and *L. fermentum* at 36 months. The latter five species typically originate from plant-based foods.

A substantial part of the solid foods prepared for babies is at risk for contamination with aflatoxins [53,54]. Food contaminated with aflatoxin B_1_ was found to affect the gut and injures the stomach and the intestine [55]. Studies carried out in animal models have shown that aflatoxin B_1_ promotes intestinal damages through perturbation of the intestinal barrier and activation of cell apoptosis and cell proliferation [55]. Saran et al. hypothesized that stunted children may fail to grow due to injury as a result of recurrent infections to the gut epithelium leading to impaired gut-mediated immunity, poor nutrient absorption, and poor appetite [56]. Thus, exposure to aflatoxin through solid foods from an early age could contribute to the high levels of stunted children observed in our cohort of 511 children [30].

In this study, the allochthonous *Lactobacillus* species isolated from the gut microbiota of Uganda children, such as *L. casei*, *L. plantarum*, *L. fermentum,* and *L. brevis* strains were shown to bind aflatoxins effectively, as also observed for these species in previous studies [57]. Therefore, it seems counterintuitive to find a significant, positive correlation for the abundance of the genus *Lactobacillus* and stunting, as the presence of this bacterial genus in the small intestine could possibly reduce the uptake of aflatoxins through binding. However, the abundance of *Lactobacillus* originating from plant-based foods may be indicative for the intake of relatively high levels of aflatoxin-contaminated foods. In contrast to the other *Lactobacillus* species, *L. salivarius* at 36 months appeared more abundant in nonstunted children. This could be related to the fact that this is a true endogenous or autochthonous *Lactobacillus* species in the human gut, and it has been shown to negatively correlate to *Shigella-*induced diarrhea in African children [58].

At this point it is not clear under which conditions binding of aflatoxin to *Lactobacillus* in the gut is most effective. Our binding assay has been performed in physiological saline with a neutral pH, which is different from the relatively low pH and other environmental conditions in the upper small intestine, where aflatoxins are absorbed [59]. As bacterial growth predominantly takes place in the colon, leading to concentrations up to 10^11^ bacteria per mL, concentrations of *Lactobacillus* in the duodenum may be too low (approximately 10^6^ bacteria mL^−1^) to effectively remove aflatoxin through binding [60,61]. Although further studies are needed to warrant their health benefits, we propose that the *Lactobacillus* species isolated from Ugandan children in this study can be further developed as locally sourced probiotics [62] and are promising candidates for decontaminating of aflatoxins through fermentation of maize-containing foods prior to consumption, as recently shown for *L. rhamnosus* [29].

## Figures and Tables

**Table 1:** Height-for-age *Z*-scores (HAZ), weight, height, lactic acid bacteria (LAB) in stool and mycotoxin concentration in urine (AFM_1_, aflatoxin M_1_), and estimated daily aflatoxin intake (AFB_1_, aflatoxin B_1_) of ten Ugandan children from Kabale and Kisoro, southwestern Uganda, at the age of 54–60 months. Values for lactic acid bacteria (LAB) in stool and AFM_1_ are means of three independent experiments. The estimated daily aflatoxin intake levels were based on measured AFB_1_ levels in maize and peanut in each subcounty (the means are presented in Figure 2).

Subject ID	District	Subcounty	Sex (M/F)	Stunted	HAZ	Weight (kg)	Height (cm)	LAB in Stool (cfu g^−1^)	AFM_1_ (pg mg^−1^)	AFB_1_ (ng kg^−1^ day^−1^)
**1634**	Kabale	Ruhija	M	YES	−4.24	12.8	90.6	8.9 × 10^6^	102	52.9
**2288**	Kisoro	Nyakabande	M	NO	−0.17	19.5	109	5.9 × 10^7^	96.1	79.4
**1553**	Kabale	Muko	F	YES	−2.64	17.0	96.2	7.4 × 10^7^	146	80.3
**2213**	Kisoro	Nyakabande	M	YES	−4.72	13.4	92.5	2.8 × 10^7^	58.7	18.5
**2439**	Kisoro	Nyarusiza	M	YES	−4.15	14.1	89.9	1.1 ×10^8^	14.8	1.1
**2112**	Kisoro	Muramba	F	NO	−0.64	17.9	106	3.8 × 10^7^	81.2	26.6
**2172**	Kisoro	Muramba	F	NO	−1.42	17.7	102	4.7 ×10^7^	168	88.6
**1317**	Kabale	Kamwganguzi	M	NO	−0.98	17.5	105	1.9 × 10^8^	110	77.0
**1161**	Kabale	Butanda	F	YES	−4.01	15.0	90.0	1.2 × 10^8^	51.2	2.3
**1434**	Kabale	Kamweesi	F	NO	0.50	18.8	112	2.9 × 10^7^	99.1	73.8

**Table 2:** *Lactobacillus* species isolated from children of Kabale and Kisoro, southwestern Uganda, aged 54–60 months with their aflatoxin B_1_ binding properties and matches in the gut microbiota at 20–24 and at 36 months presented as percentage abundance and prevalence. Values for bound aflatoxin B_1_ (%) are means ± standard deviations of three independent experiments at a cell concentration of 10^8^ cfu mL^−1^.

Subject ID	Identity * (%)	Isolate **	Bound Aflatoxin B_1_ (%)	Abundance (%) ***		Prevalence (%) ***	
				20–24 Months	36 Months	20–24 Months	36 Months
**1634**	**100**	***L. plantarum*** **APW1634**	34.3 ± 6.7	0.003	0.7	15	45
**2288**	99	*L. fermentum* APW2288	25.1 ± 0.4	0.03	0.2	22.9	34.3
**2288**	99	*L. rhamnosus* APW2288B	13.7 ± 0	0	0.007	0	2.1
**2288**	99	*L. casei* APW2288E	59.6 ± 6.3	0.0005	0.1	3.6	17.9
**1553**	99	*L. plantarum* APW1553A	19.2 ± 0	0.003	0.7	15	45
**1553**	99	*L. brevis* APW1553	45.9 ± 3.1	0.003	0.7	7.9	30
**2213**	99	*L. casei* APW2213	62.6 ± 4.8	0.0005	0.1	3.6	17.9
**2213**	99	*L. buchneri* APW2213E	42.1 ± 8.2	0.0002	0.1	2.1	16.4
**2439**	99	*L. casei* APW2439C	37.9 ± 11.1	0.0005	0.1	3.6	17.9
**2439**	96	*L. casei* APW2439A	35.4 ± 5.5	0.0005	0.1	3.6	17.9
**2112**	99	*L. plantarum* APW2112A	0.9 ± 1.3	0.003	0.7	15	45
**2112**	99	*L. brevis* APW2112	31.8 ± 0.7	0.003	0.7	7.9	30
**2112**	99	*L. casei* APW2112D	46.9 ± 1.5	0.0005	0.1	3.6	17.9
**2172**	99	*L. casei* APW2172A	20.1 ± 0	0.0005	0.1	3.6	17.9
**2172**	99	*L. casei* APW2172C	49.1 ± 3.9	0.0005	0.1	3.6	17.9
**1317**	99	*L. plantarum* APW1317A	49.4 ± 14.7	0.003	0.7	15	45
**1317**	99	*L. fermentum* APW1317	76.1 ± 12.9	0.03	0.2	22.9	34.3
**1161**	99	*L. casei* APW1161	54.5 ± 2.8	0.0005	0.1	3.6	17.9
**1161**	99	*L. pantheris* APW1161C	57.6 ± 1.7	0	0.003	0	4.3
**1161**	99	*L. paracasei* APW1161D	46.7 ± 17.7	0.0005	0.1	3.6	17.9
**1434**	99	*L. plantarum* APW1434B	10.7 ± 0	0.003	0.7	15	45
**1434**	99	*L. fermentum* APW1434	45.9 ± 9.3	0.03	0.2	22.9	34.3
**1434**	99	*L. casei* APW1434D	25.9 ± 0.7	0.0005	0.1	3.6	17.9

* Identity value is based on the match of the sequenced nearly full-length 16S rRNA gene of the *Lactobacillus* isolate with the 16S rRNA sequence from GenBank. ** *Lactobacillus* isolates with unique APW strain coding were assigned to species with distinct 16S rRNA gene sequences. *** Abundance and prevalence values were based on 100% identity matches of the unique V4 regions of the sequenced 16S rRNA genes of the isolates with the V4 sequences present in the gut microbiota data of the cohort of rural Uganda children. Values cannot be unambiguously assigned to *Lactobacillus* strains and are only a partial representation of the *Lactobacillus* species in the gut microbiota.

## Supplementary Materials

The following are available online at https://www.mdpi.com/2076-2607/8/3/347/s1, File S1: The frequency of consumption of a particular food type for southwestern Ugandans, File S2: The limit of detection (LOD), precision, and accuracy of the immunoassays used for aflatoxin determination in urine and food samples, File S3: Collection 16S rRNA gene sequences for *Lactobacillus* species identification, File S4: PERMANOVA correlation coefficients and *p*-values.
