# Supplementary material for: Aflatoxins: Occurrence, Exposure, and Binding to Lactobacillus Species from the Gut Microbiota of Rural Ugandan Children
Source: Microorganisms. 2020 Feb 29;8(3):347. doi: 10.3390/microorganisms8030347 (PMC7143030; doi:10.3390/microorganisms8030347)
Supplement: Supplementary file 1 [file microorganisms-08-00347-s001.zip › suppl files/Suplementary file S2 Validation of aflatoxin immunoassay.docx]

**The results for the validation of the immunoassays**

| **Urine** | | |
| --- | --- | --- |
| Limit of Detection | 5.8 ng l^-1^ | |
|  | Aflatoxin M1 in urine (ppt) | CV (%) |
| Precision (CV/%) | 125 | 5.2 |
|  | 1000 | 6.6 |
|  | 2000 | 7.2 |
|  |  |  |
|  | Aflatoxin M1 in urine (ppt) | Recovery (%) |
| Recovery (%) | 125 | 82 |
|  | 1000 | 94 |
|  | 2000 | 73.8 |
| **Maize flour** | | |
| Limit of detection | 1.8 |  |
|  |  |  |
|  | Aflatoxin B_1_ in maize flour (µg l^-1^) | CV (%) |
| Precision (CV/%) | 1 | 8.6 |
|  | 10 | 4.9 |
|  | 50 | 5.8 |
|  |  |  |
| Accuracy | Aflatoxin B_1_ in maize flour (µg l^-1^) | Recovery (%) |
| Recovery (%) | 1 | 67.2 |
|  | 10 | 73.9 |
|  | 50 | 70.6 |
| **Peanut** | | |
|  | Aflatoxin B_1_ in peanut (µg l^-1^) | CV (%) |
| Precision (CV/%) | 1 | 3.1 |
|  | 10 | 5.4 |
|  | 50 | 3.6 |
|  |  |  |
| Accuracy | Aflatoxin B_1_ in peanut (µg l^-1^) | Recovery (%) |
| Recovery (%) | 1 | 77.1 |
|  | 10 | 50.7 |
|  | 50 | 57 |
|  |  |  |

Limit of detection, accuracy and recovery were calculated as described by Wacoo et al., [[1](#_ENREF_1)]. Briefly, the immunosensor was calibrated by analyzing aflatoxin B_1_ standard (0, 1, 5, 10, 20 and 50 µg l^-1^). For determination of limit of detection, the correlation coefficient and slope of the regression line of each curve were determined.

The limit of detection (LOD) was expressed as:

LOD = (3.3 ∗ mean of standard deviation of 4 calibration curve)/average slope of 4 calibration curve

The precision was calculated as the degree of scatter (coefficient of variation) between four measurements of aflatoxin B_1_ Standards. The accuracy was estimated from the recovery for the three different aflatoxin levels.

**Reference**

1. Wacoo, P.A.; Ocheng, M.; Wendiro, D.; Vuzi, P.C.; Hawumba, F.J. Development and characterization of an electroless plated silver/cysteine sensor platform for the electrochemical determination of aflatoxin b1. . *Journal of Sensors* **2016**, *2015*, 1-8.
