# Supplementary material for: Aflatoxins: Occurrence, Exposure, and Binding to Lactobacillus Species from the Gut Microbiota of Rural Ugandan Children
Source: Microorganisms. 2020 Feb 29;8(3):347. doi: 10.3390/microorganisms8030347 (PMC7143030; doi:10.3390/microorganisms8030347)
Supplement: Supplementary file 1 [file microorganisms-08-00347-s001.zip › suppl files/Supplementary file S1 Food frequency questionaire and table.docx]

**Food frequency Questionnaire**

**Demographic characteristics**

**District 1=Kabale 2=Kisoro**

**Sub-county………………………………………………..Village…………………….**

**Household IDNO…………………………**

**Interview date __ __/ __ __ /__ __ __ __ (day month year);**

**I would like to know how often you consume the following foods.**

Posho and/ porridge

Dry maize with beans (empengyeri)

Beans

Pea nuts/ Ground nuts

Sorghum (Kyankalata)

Sorghum porridge (enkumba)

Millet porridge

Sprouted sorghum porridge (Bushera /Musururu)

Rice

Wheat

Cassava

Are there any other foods that you normally consume (not listed above 1= Yes 2= No

**If Yes, List them and give the frequency of consumption**

1. **…………………….**
2. **…………………….**
3. **……………………**
4. **……………………**

**Responses**

1= Three times a day 6= 4 times a month

2= Two times a day 7= 2-3 times a month

3= Once a day 8= Rarely

4= 5-6 times a week 9= Never

5= 1-4 times a week

**Anthropometry for the child**

| Weight 1 (kg) |  | Weight 2 (kg ) |  | Average weight (kg) |  |
| --- | --- | --- | --- | --- | --- |
| Length/ height 1 (cm) |  | Length/ height 2 (cm) |  | Average Length/ height (cm) |  |
| MUAC 1 (cm) |  | MUAC 2 (cm) |  | Average MUAC (cm) |  |

24. Observe for clinical signs: **Present = 1 Absent = 2**

- Anaemia

- Oedema
- Skin rash
- Light hair
- Buggy pants

Table S3. The frequency of commonly consumed food in South-Western Uganda, district of Kisoro and Kable on a on a 9-point scale: 1= Three times a day, 2= Two times a day, 3= Once a day, 4= 5-6 times a week, 5= 1-4 times a week, 6= 4 times a month, 7= 2-3 times a month, 8= Rarely and 9= Never (Values are means + SD, n = 22).

| Beans | 2 ± 0.6 |
| --- | --- |
| Peanuts | 6 ± 1.6 |
| Sorghum Bread | 7.8 ± 2 |
| Sorghum porridge | 5.8 ± 2.5 |
| millet porridge | 6.4 ± 2.5 |
| Fermented porridge | 4.5 ± 2.6 |
| Rice | 7.4 ± 1.9 |
| Wheat | 8.2 ± 2 |
| Cassava | 7.4 ± 2.2 |
| Irish Potatoes | 3.6 ± 1.9 |
| Sweet potatoes | 4.2 ± 1.7 |
| Peas | 4.9 ± 1.7 |
| Greens (*eshiga*) | 3.3 ± 1.7 |
