# Supplementary material for: Aflatoxins: Occurrence, Exposure, and Binding to Lactobacillus Species from the Gut Microbiota of Rural Ugandan Children
Source: Microorganisms. 2020 Feb 29;8(3):347. doi: 10.3390/microorganisms8030347 (PMC7143030; doi:10.3390/microorganisms8030347)
Supplement: Supplementary file 1 [file microorganisms-08-00347-s001.zip › suppl files/Supplementary file S3 16S rRNA sequences.rtf]

>Lactobacillus plantarum  APW1317A
TGTCACTTAGGCGGCTGGTTCCTAAAAGGTTACCCCACCGACTTTGGGTGTTACAAACTCTCATGGTGTGACGGGCGGTGTGTACAAGGCCCGGGAACGTATTCACCGCGGCATGCTGATCCGCGATTACTAGCGATTCCGACTTCATGTAGGCGAGTTGCAGCCTACAATCCGAACTGAGAATGGCTTTAAGAGATTAGCTTACTCTCGCGAGTTCGCAACTCGTTGTACCATCCATTGTAGCACGTGTGTAGCCCAGGTCATAAGGGGCATGATGATTTGACGTCATCCCCACCTTCCTCCGGTTTGTCACCGGCAGTCTCACCAGAGTGCCCAACTTAATGCTGGCAACTGATAATAAGGGTTGCGCTCGTTGCGGGACTTAACCCAACATCTCACGACACGAGCTGACGACAACCATGCACCACCTGTATCCATGTCCCCGAAGGGAACGTCTAATCTCTTAGATTTGCATAGTATGTCAAGACCTGGTAAGGTTCTTCGCGTAGCTTCGAATTAAACCACATGTTCCACCGCTTGTGCGGGCCCCCGTCAATTCCTTTGAGTTTCAGCCTTGCGGCCGTACTCCCCAGGCGGAATGCTTAATGCGTTAGCTGCAGCACTGAAGGGCGGAAACCCTCCAACACTTAGCATTCATCGTTTACGGTATGGACTACCAGGGTATCTAATCCTGTTTGCTACCCATACTTTCGAGCCTCAGCGTCAGTTACAGACCAGACAGCCGCCTTCGCCACTGGTGTTCTTCCATATATCTACGCATTTCACCGCTACACATGGAGTTCCACTGTCCTCTTCTGCACTCAAGTTTCCCAGTTTCCGATGCACTTCTTCGGTTGAGCCGAAGGCTTTCACATCAGACTTAAAAAACCGCCTGCGCTCGCTTTACGCCCAATAAATCCGGACAACGCTTGCCACCTACGTATTACCGCGGCTGCTGGCACGTAGTTAGCCGTGGCTTTCTGGTTAAATACCGTCAATACCTGAACAGTTACTCTCAGATATGTTCTTCTTTAACAACAGAGTTTTACGAGCCGAAACCCTTCTTCACTCACGGGGCGTTGCTCCATCAGACTTTCGTCCATTGTGGAAGATTCCCTACTGCTGCCTCCCGTAGGAGTTTGGGCCGTGTCTCAGTCCCAATGTGGCCGATTACCCTCTCAGGTCGGCTACGTATCATTGCCATGGTGAGCCGTTACCCCACCATCTAGCTAATACGCCGCGGGACCATCCAAAAGTGATAGCCGAAGCCATCTTTCAAACTCGGACCCTGCGGTCCAAGTTGTTATGCGGTATTAGCATCTGTTTCCAGGTGTTATCCCCCGCTTCTGGGCAGGTTTCCCACGTGTTACTCACCAGTTCGCCACTCACTCAAATGTAAATCATGATGCAAGCACCAATCAATACCAGGAGTTCGTCGACTGCATGTATAGGCAC

>Lactobacillus fermentum APW1434C
CTTAGGCGGCTGGCTCCTAAAGGTTACCCCACCGACTTTGGGTGTTACAAACTCTCATGGTGTGACGGGCGGTGTGTACAAGGCCCGGGAACGTATTCACCGCGGCATGCTGATCCGCGATTACTAGCGATTCCGACTTCGTGCAGGCGAGTTGCAGCCTGCAGTCCGAACTGAGAACGGTTTTAAGAGATTTGCTTGCCCTCGCGAGTTCGCGACTCGTTGTACCGTCCATTGTAGCACGTGTGTAGCCCAGGTCATAAGGGGCATGATGATCTGACGTCGTCCCCACCTTCCTCCGGTTTGTCACCGGCAGTCTCACTAGAGTGCCCAACTTAATGCTGGCAACTAGTAACAAGGGTTGCGCTCGTTGCGGGACTTAACCCAACATCTCACGACACGAGCTGACGACGACCATGCACCACCTGTCATTGCGTTCCCGAAGGAAACGCCCTATCTCTAGGGTTGGCGCAAGATGTCAAGACCTGGTAAGGTTCTTCGCGTAGCTTCGAATTAAACCACATGCTCCACCGCTTGTGCGGGCCCCCGTCAATTCCTTTGAGTTTCAACCTTGCGGTCGTACTCCCCAGGCGGAGTGCTTAATGCGTTAGCTCCGGCACTGAAGGGCGGAAACCCTCCAACACCTAGCACTCATCGTTTACGGCATGGACTACCAGGGTATCTAATCCTGTTCGCTACCCATGCTTTCGAGTCTCAGCGTCAGTTGCAGACCAGGTAGCCGCCTTCGCCACTGGTGTTCTTCCATATATCTACGCATTCCACCGCTACACATGGAGTTCCACTACCCTCTTCTGCACTCAAGTTATCCAGTTTCCGATGCACTTCTCCGGTTAAGCCGAAGGCTTTCACATCAGACTTAGAAAACCGCCTGCACTCTCTTTACGCCCAATAAATCCGGATAACGCTTGCCACCTACGTATTACCGCGGCTGCTGGCACGTAGTTAGCCGTGACTTTCTGGTTAAATACCGTCAACGTATGAACAGTTACTCTCATACGTGTTCTTCTTTAACAACAGAGCTTTACGAGCCGAAACCCTTCTTCACTCACGCGGTGTTGCTCCATCAGGCTTGCGCCCATTGTGGAAGATTCCCTACTGCTGCCTCCCGTAGGAGTATGGGCCGTGTCTCAGTCCCATTGTGGCCGATCAGTCTCTCAACTTGGCTATGCATCATCGCCTTGGTAGGCCGTTACCCCACCAACAAGCTAATGCACCGCAGGTCCATCCAGAAGTGATAGCGAGAAGCCATCTTTTAAGCGTTGTTCATGCGAACAACGTTGTTATGCGGTATTAGCATCTGTTTCCAAATGTTGTCCCCCGCTTCTGGGCAGGTTACCTACGTGTTACTCACCCGTCCGCCACTCGTTGGCGACCAAAATCAATCAGGTGCAAGCACCATCAATCAATTGGGCCAACGCGTTCGACTGCATGTATAGGCACCG

>Lactobacillus casei APW2213D
CACTTAGACGGCTCGCTCCCTAAAGGGTTACGCCACCGGCTTCGGGTGTTACAAACTCTCATGGTGTGACGGGCGGTGTGTACAAGGCCCGGGAACGTATTCACCGCGGCGTGCTGATCCGCGATTACTAGCGATTCCGACTTCGTGTAGGCGAGTTGCAGCCTACAGTCCGAACTGAGAATGGCTTTAAGAGATTAGCTTGACCTCGCGGTCTCGCAACTCGTTGTACCATCCATTGTAGCACGTGTGTAGCCCAGGTCATAAGGGGCATGATGATTTGACGTCATCCCCACCTTCCTCCGGTTTGTCACCGGCAGTCTTACTAGAGTGCCCAACTAAATGCTGGCAACTAGTCATAAGGGTTGCGCTCGTTGCGGGACTTAACCCAACATCTCACGACACGAGCTGACGACAACCATGCACCACCTGTCATTTTGCCCCCGAAGGGGAAACCTGATCTCTCAGGTGATCAAAAGATGTCAAGACCTGGTAAGGTTCTTCGCGTTGCTTCGAATTAAACCACATGCTCCACCGCTTGTGCGGGCCCCCGTCAATTCCTTTGAGTTTCAACCTTGCGGTCGTACTCCCCAGGCGGAATGCTTAATGCGTTAGCTGCGGCACTGAAGGGCGGAAACCCTCCAACACCTAGCATTCATCGTTTACGGCATGGACTACCAGGGTATCTAATCCTGTTCGCTACCCATGCTTTCGAGCCTCAGCGTCAGTTACAGACCAGACAGCCGCCTTCGCCACTGGTGTTCTTCCATATATCTACGCATTTCACCGCTACACATGGAGTTCCACTGTCCTCTTCTGCACTCAAGTTTCCCAGTTTCCGATGCGCTTCCTCGGTTAAGCCGAGGGCTTTCACATCAGACTTAAAAAACCGCCTGCGCTCGCTTTACGCCCAATAAATCCGGATAACGCTTGCCACCTACGTATTACCGCGGCTGCTGGCACGTAGTTAGCCGTGGCTTTCTGGTTGGATACCGTCACGCCGACAACAGTTACTCTGCCGACCATTCTTCTCCAACAACAGAGTTTTACGACCCGAAAGCCTTCTTCACTCACGCGGCGTTGCTCCATCAGACTTGCGTCCATTGTGGAAGATTCCCTACTGCTGCCTCCCGTAGGAGTTTGGGCCGTGTCTCAGTCCCAATGTGGCCGATCAACCTCTCAGTTTGGCTACGTATCATCGCCTTGGTGAGCCATTACCTCACCAACTAGCTAATACGCCGCGGGTCCATCCAAAAGCGATAGCTTACGCCATCTTTCAGCCAAGAACCATGCGGTTCTTGGATCTATGCGGTATTAGCATCTGTTTCCAAATGTTATCCCCCACTTAAGGGCAGGTTACCCACGTGTTACTCACCCGTCCGCCACTCGTTCCATGTTGAATCTCGGTGCAAGCACCGATCATCAACGAGAACTCGTTCGACTGCATGTATAGG

>Lactobacillus casei APW2213C 
GTCACTTAGACGGCTCGCTCCTAAAGGGTTACGCCACCGGCTTCGGGTGTTACAAACTCTCATGGTGTGACGGGCGGTGTGTACAAGGCCCGGGAACGTATTCACCGCGGCGTGCTGATCCGCGATTACTAGCGATTCCGACTTCGTGTAGGCGAGTTGCAGCCTACAGTCCGAACTGAGAATGGCTTTAAGAGATTAGCTTGACCTCGCGGTCTCGCAACTCGTTGTACCATCCATTGTAGCACGTGTGTAGCCCAGGTCATAAGGGGCATGATGATTTGACGTCATCCCCACCTTCCTCCGGTTTGTCACCGGCAGTCTTACTAGAGTGCCCAACTAAATGCTGGCAACTAGTCATAAGGGTTGCGCTCGTTGCGGGACTTAACCCAACATCTCACGACACGAGCTGACGACAACCATGCACCACCTGTCATTTTGCCCCCGAAGGGGAAACCTGATCTCTCAGGTGATCAAAAGATGTCAAGACCTGGTAAGGTTCTTCGCGTTGCTTCGAATTAAACCACATGCTCCACCGCTTGTGCGGGCCCCCGTCAATTCCTTTGAGTTTCAACCTTGCGGTCGTACTCCCCAGGCGGAATGCTTAATGCGTTAGCTGCGGCACTGAAGGGCGGAAACCCTCCAACACCTAGCATTCATCGTTTACGGCATGGACTACCAGGGTATCTAATCCTGTTCGCTACCCATGCTTTCGAGCCTCAGCGTCAGTTACAGACCAGACAGCCGCCTTCGCCACTGGTGTTCTTCCATATATCTACGCATTTCACCGCTACACATGGAGTTCCACTGTCCTCTTCTGCACTCAAGTTTCCCAGTTTCCGATGCGCTTCCTCGGTTAAGCCGAGGGCTTTCACATCAGACTTAAAAAACCGCCTGCGCTCGCTTTACGCCCAATAAATCCGGATAACGCTTGCCACCTACGTATTACCGCGGCTGCTGGCACGTAGTTAGCCGTGGCTTTCTGGTTGGATACCGTCACGCCGACAACAGTTACTCTGCCGACCATTCTTCTCCAACAACAGAGTTTTACGACCCGAAAGCCTTCTTCACTCACGCGGCGTTGCTCCATCAGACTTGCGTCCATTGTGGAAGATTCCCTACTGCTGCCTCCCGTAGGAGTTTGGGCCGTGTCTCAGTCCCAATGTGGCCGATCAACCTCTCAGTTCGGCTACGTATCATCGCCTTGGTGAGCCATTACCTCACCAACTAGCTAATACGCCGCGGGTCCATCCAAAAGCGATAGCTTACGCCATCTTTCAGCCAAGAACCATGCGGTTCTTGGATCTATGCGGTATTAGCATCTGTTTCCAAATGTTATCCCCCACTTAAGGGCAGGTTACCCACGTGTTACTCACCCGTCCGCCACTCGTTCCATGTTGAATCTCGGTGCAAGCACCGATCATCAACGAGAACTCGTTCGACTGCATGTATAGCAGC

>Lactobacillus casei APW2213B
CCACTTAGACGGCTCGCTCCTAAAGGGTTACGCCACCGGCTTCGGGTGTTACAAACTCTCATGGTGTGACGGGCGGTGTGTACAAGGCCCGGGAACGTATTCACCGCGGCGTGCTGATCCGCGATTACTAGCGATTCCGACTTCGTGTAGGCGAGTTGCAGCCTACAGTCCGAACTGAGAATGGCTTTAAGAGATTAGCTTGACCTCGCGGTCTCGCAACTCGTTGTACCATCCATTGTAGCACGTGTGTAGCCCAGGTCATAAGGGGCATGATGATTTGACGTCATCCCCACCTTCCTCCGGTTTGTCACCGGCAGTCTTACTAGAGTGCCCAACTAAATGCTGGCAACTAGTCATAAGGGTTGCGCTCGTTGCGGGACTTAACCCAACATCTCACGACACGAGCTGACGACAACCATGCACCACCTGTCATTTTGCCCCCGAAGGGGAAACCTGATCTCTCAGGTGATCAAAAGATGTCAAGACCTGGTAAGGTTCTTCGCGTTGCTTCGAATTAAACCACATGCTCCACCGCTTGTGCGGGCCCCCGTCAATTCCTTTGAGTTTCAACCTTGCGGTCGTACTCCCCAGGCGGAATGCTTAATGCGTTAGCTGCGGCACTGAAGGGCGGAAACCCTCCAACACCTAGCATTCATCGTTTACGGCATGGACTACCAGGGTATCTAATCCTGTTCGCTACCCATGCTTTCGAGCCTCAGCGTCAGTTACAGACCAGACAGCCGCCTTCGCCACTGGTGTTCTTCCATATATCTACGCATTTCACCGCTACACATGGAGTTCCACTGTCCTCTTCTGCACTCAAGTTTCCCAGTTTCCGATGCGCTTCCTCGGTTAAGCCGAGGGCTTTCACATCAGACTTAAAAAACCGCCTGCGCTCGCTTTACGCCCAATAAATCCGGATAACGCTTGCCACCTACGTATTACCGCGGCTGCTGGCACGTAGTTAGCCGTGGCTTTCTGGTTGGATACCGTCACGCCGACAACAGTTACTCTGCCGACCATTCTTCTCCAACAACAGAGTTTTACGACCCGAAAGCCTTCTTCACTCACGCGGCGTTGCTCCATCAGACTTGCGTCCATTGTGGAAGATTCCCTACTGCTGCCTCCCGTAGGAGTTTGGGCCGTGTCTCAGTCCCAATGTGGCCGATCAACCTCTCAGTTTGGCTACGTATCATCGCCTTGGTGAGCCATTACCTCACCAACTAGCTAATACGCCGCGGGTCCATCCAAAAGCGATAGCTTACGCCATCTTTCAGCCAAGAACCATGCGGTTCTTGGATCTATGCGGTATTAGCATCTGTTTCCAAATGTTATCCCCCACTTAAGGGCAGGTTACCCACGTGTTACTCACCCGTCCGCCACTCGTTCCATGTTGAATCTCGGTGCAAGCACCGATCATCAACGAGAACTCGTTCGACTGCATGTATAGGA

>Lactobacillus casei APW2213A 
CACTTAGACGGCTCGCTCCCTAAAGGGTTACGCCACCGGCTTCGGGTGTTACAAACTCTCATGGTGTGACGGGCGGTGTGTACAAGGCCCGGGAACGTATTCACCGCGGCGTGCTGATCCGCGATTACTAGCGATTCCGACTTCGTGTAGGCGAGTTGCAGCCTACAGTCCGAACTGAGAATGGCTTTAAGAGATTAGCTTGACCTCGCGGTCTCGCAACTCGTTGTACCATCCATTGTAGCACGTGTGTAGCCCAGGTCATAAGGGGCATGATGATTTGACGTCATCCCCACCTTCCTCCGGTTTGTCACCGGCAGTCTTACTAGAGTGCCCAACTAAATGCTGGCAACTAGTCATAAGGGTTGCGCTCGTTGCGGGACTTAACCCAACATCTCACGACACGAGCTGACGACAACCATGCACCACCTGTCATTTTGCCCCCGAAGGGGAAACCTGATCTCTCAGGTGATCAAAAGATGTCAAGACCTGGTAAGGTTCTTCGCGTTGCTTCGAATTAAACCACATGCTCCACCGCTTGTGCGGGCCCCCGTCAATTCCTTTGAGTTTCAACCTTGCGGTCGTACTCCCCAGGCGGAATGCTTAATGCGTTAGCTGCGGCACTGAAGGGCGGAAACCCTCCAACACCTAGCATTCATCGTTTACGGCATGGACTACCAGGGTATCTAATCCTGTTCGCTACCCATGCTTTCGAGCCTCAGCGTCAGTTACAGACCAGACAGCCGCCTTCGCCACTGGTGTTCTTCCATATATCTACGCATTTCACCGCTACACATGGAGTTCCACTGTCCTCTTCTGCACTCAAGTTTCCCAGTTTCCGATGCGCTTCCTCGGTTAAGCCGAGGGCTTTCACATCAGACTTAAAAAACCGCCTGCGCTCGCTTTACGCCCAATAAATCCGGATAACGCTTGCCACCTACGTATTACCGCGGCTGCTGGCACGTAGTTAGCCGTGGCTTTCTGGTTGGATACCGTCACGCCGACAACAGTTACTCTGCCGACCATTCTTCTCCAACAACAGAGTTTTACGACCCGAAAGCCTTCTTCACTCACGCGGCGTTGCTCCATCAGACTTGCGTCCATTGTGGAAGATTCCCTACTGCTGCCTCCCGTAGGAGTTTGGGCCGTGTCTCAGTCCCAATGTGGCCGATCAACCTCTCAGTTCGGCTACGTATCATCGCCTTGGTGAGCCATTACCTCACCAACTAGCTAATACGCCGCGGGTCCATCCAAAAGCGATAGCTTACGCCATCTTTCAGCCAAGAACCCTGCGGTTCTTGGATCTTTGCGGTATTAGCATCTGTTTCCAAATGTTATCCCCCACTTAAGGGCAGGTTACCCACGTGTTACTCACCCGTCCGCCACTCGTTCCATGTTGAATCTCGGTGCAAGCACCGATCATCAACGAGAACTCGTTCGACTTGCATGTATAGC

> Lactobacillus casei APW2172E
GTCACTTAGACGGCTCGCTCCCTAAAAGGGTTACGCCACCGGCTTCGGGTGTTACAAACTCTCATGGTGTGACGGGCGGTGTGTACAAGGCCCGGGAACGTATTCACCGCGGCGTGCTGATCCGCGATTACTAGCGATTCCGACTTCGTGTAGGCGAGTTGCAGCCTACAGTCCGAACTGAGAATGGCTTTAAGAGATTAGCTTGACCTCGCGGTCTCGCAACTCGTTGTACCATCCATTGTAGCACGTGTGTAGCCCAGGTCATAAGGGGCATGATGATTTGACGTCATCCCCACCTTCCTCCGGTTTGTCACCGGCAGTCTTACTAGAGTGCCCAACTAAATGCTGGCAACTAGTCATAAGGGTTGCGCTCGTTGCGGGACTTAACCCAACATCTCACGACACGAGCTGACGACAACCATGCACCACCTGTCATTTTGCCCCCGAAGGGGAAACCTGATCTCTCAGGTGATCAAAAGATGTCAAGACCTGGTAAGGTTCTTCGCGTTGCTTCGAATTAAACCACATGCTCCACCGCTTGTGCGGGCCCCCGTCAATTCCTTTGAGTTTCAACCTTGCGGTCGTACTCCCCAGGCGGAATGCTTAATGCGTTAGCTGCGGCACTGAAGGGCGGAAACCCTCCAACACCTAGCATTCATCGTTTACGGCATGGACTACCAGGGTATCTAATCCTGTTCGCTACCCATGCTTTCGAGCCTCAGCGTCAGTTACAGACCAGACAGCCGCCTTCGCCACTGGTGTTCTTCCATATATCTACGCATTTCACCGCTACACATGGAGTTCCACTGTCCTCTTCTGCACTCAAGTTTCCCAGTTTCCGATGCGCTTCCTCGGTTAAGCCGAGGGCTTTCACATCAGACTTAAAAAACCGCCTGCGCTCGCTTTACGCCCAATAAATCCGGATAACGCTTGCCACCTACGTATTACCGCGGCTGCTGGCACGTAGTTAGCCGTGGCTTTCTGGTTGGATACCGTCACGCCGACAACAGTTACTCTGCCGACCATTCTTCTCCAACAACAGAGTTTTACGACCCGAAAGCCTTCTTCACTCACGCGGCGTTGCTCCATCAGACTTGCGTCCATTGTGGAAGATTCCCTACTGCTGCCTCCCGTAGGAGTTTGGGCCGTGTCTCAGTCCCAATGTGGCCGATCAACCTCTCAGTTTGGCTACGTATCATCGCCTTGGTGAGCCATTACCTCACCAACTAGCTAATACGCCGCGGGTCCATCCAAAAGCGATAGCTTACGCCATCTTTCAGCCAAGAACCATGCGGTTCTTGGATCTATGCGGTATTAGCATCTGTTTCCAAATGTTATCCCCCACTTAAGGGCAGGTTACCCACGTGTTACTCACCCGTCCGCCACTCGTTCCATGTTGAATCTCGGTGCAAGCACCGATCATCAACGAGAACTCGTTCGACTGCATGTATAGGCACGCCG

>Lactobacillus casei APW2172D
GTCACTTAGACGGCTCGCTCCCTAAAAGGGTTACGCCACCGGCTTCGGGTGTTACAAACTCTCATGGTGTGACGGGCGGTGTGTACAAGGCCCGGGAACGTATTCACCGCGGCGTGCTGATCCGCGATTACTAGCGATTCCGACTTCGTGTAGGCGAGTTGCAGCCTACAGTCCGAACTGAGAATGGCTTTAAGAGATTAGCTTGACCTCGCGGTCTCGCAACTCGTTGTACCATCCATTGTAGCACGTGTGTAGCCCAGGTCATAAGGGGCATGATGATTTGACGTCATCCCCACCTTCCTCCGGTTTGTCACCGGCAGTCTTACTAGAGTGCCCAACTAAATGCTGGCAACTAGTCATAAGGGTTGCGCTCGTTGCGGGACTTAACCCAACATCTCACGACACGAGCTGACGACAACCATGCACCACCTGTCATTTTGCCCCCGAAGGGGAAACCTGATCTCTCAGGTGATCAAAAGATGTCAAGACCTGGTAAGGTTCTTCGCGTTGCTTCGAATTAAACCACATGCTCCACCGCTTGTGCGGGCCCCCGTCAATTCCTTTGAGTTTCAACCTTGCGGTCGTACTCCCCAGGCGGAATGCTTAATGCGTTAGCTGCGGCACTGAAGGGCGGAAACCCTCCAACACCTAGCATTCATCGTTTACGGCATGGACTACCAGGGTATCTAATCCTGTTCGCTACCCATGCTTTCGAGCCTCAGCGTCAGTTACAGACCAGACAGCCGCCTTCGCCACTGGTGTTCTTCCATATATCTACGCATTTCACCGCTACACATGGAGTTCCACTGTCCTCTTCTGCACTCAAGTTTCCCAGTTTCCGATGCGCTTCCTCGGTTAAGCCGAGGGCTTTCACATCAGACTTAAAAAACCGCCTGCGCTCGCTTTACGCCCAATAAATCCGGATAACGCTTGCCACCTACGTATTACCGCGGCTGCTGGCACGTAGTTAGCCGTGGCTTTCTGGTTGGATACCGTCACGCCGACAACAGTTACTCTGCCGACCATTCTTCTCCAACAACAGAGTTTTACGACCCGAAAGCCTTCTTCACTCACGCGGCGTTGCTCCATCAGACTTGCGTCCATTGTGGAAGATTCCCTACTGCTGCCTCCCGTAGGAGTTTGGGCCGTGTCTCAGTCCCAATGTGGCCGATCAACCTCTCAGTTCGGCTACGTATCATCGCCTTGGTGAGCCATTACCTCACCAACTAGCTAATACGCCGCGGGTCCATCCAAAAGCGATAGCTTACGCCATCTTTCAGCCAAGAACCATGCGGTTCTTGGATCTATGCGGTATTAGCATCTGTTTCCAAATGTTATCCCCCACTTAAGGGCAGGTTACCCACGTGTTACTCACCCGTCCGCCACTCGTTCCATGTTGAATCTCGGTGCAAGCACCGATCATCAACGAGAACTCGTCGACTGCATGTATAGGCACG

>Lactobacillus casei APW2172C
TGTCACCTTAGACGGCTCGCTCCCTAAAGGGTTACGCCACCGGCTTCGGGTGTTACAAACTCTCATGGTGTGACGGGCGGTGTGTACAAGGCCCGGGAACGTATTCACCGCGGCGTGCTGATCCGCGATTACTAGCGATTCCGACTTCGTGTAGGCGAGTTGCAGCCTACAGTCCGAACTGAGAATGGCTTTAAGAGATTAGCTTGACCTCGCGGTCTCGCAACTCGTTGTACCATCCATTGTAGCACGTGTGTAGCCCAGGTCATAAGGGGCATGATGATTTGACGTCATCCCCACCTTCCTCCGGTTTGTCACCGGCAGTCTTACTAGAGTGCCCAACTAAATGCTGGCAACTAGTCATAAGGGTTGCGCTCGTTGCGGGACTTAACCCAACATCTCACGACACGAGCTGACGACAACCATGCACCACCTGTCATTTTGCCCCCGAAGGGGAAACCTGATCTCTCAGGTGATCAAAAGATGTCAAGACCTGGTAAGGTTCTTCGCGTTGCTTCGAATTAAACCACATGCTCCACCGCTTGTGCGGGCCCCCGTCAATTCCTTTGAGTTTCAACCTTGCGGTCGTACTCCCCAGGCGGAATGCTTAATGCGTTAGCTGCGGCACTGAAGGGCGGAAACCCTCCAACACCTAGCATTCATCGTTTACGGCATGGACTACCAGGGTATCTAATCCTGTTCGCTACCCATGCTTTCGAGCCTCAGCGTCAGTTACAGACCAGACAGCCGCCTTCGCCACTGGTGTTCTTCCATATATCTACGCATTTCACCGCTACACATGGAGTTCCACTGTCCTCTTCTGCACTCAAGTTTCCCAGTTTCCGATGCGCTTCCTCGGTTAAGCCGAGGGCTTTCACATCAGACTTAAAAAACCGCCTGCGCTCGCTTTACGCCCAATAAATCCGGATAACGCTTGCCACCTACGTATTACCGCGGCTGCTGGCACGTAGTTAGCCGTGGCTTTCTGGTTGGATACCGTCACGCCGACAACAGTTACTCTGCCGACCATTCTTCTCCAACAACAGAGTTTTACGACCCGAAAGCCTTCTTCACTCACGCGGCGTTGCTCCATCAGACTTGCGTCCATTGTGGAAGATTCCCTACTGCTGCCTCCCGTAGGAGTTTGGGCCGTGTCTCAGTCCCAATGTGGCCGATCAACCTCTCAGTTCGGCTACGTATCATCGCCTTGGTGAGCCATTACCTCACCAACTAGCTAATACGCCGCGGGTCCATCCAAAAGCGATAGCTTACGCCATCTTTCAGCCAAGAACCATGCGGTTCTTGGATCTATGCGGTATTAGCATCTGTTTCCAAATGTTATCCCCCACTTAAGGGCAGGTTACCCACGTGTTACTCACCCGTCCGCCACTCGTTCCATGTTGAATCTCTGGTGCAAGCACCGATCATCAACGAGAACTCGTCGACTGCATGTATAGGACGC

>Lactobacillus casei APW2172B
TTGTCACTTAGACGGCTCGCTCCCTAAAGGGTTACGCCACCGGCTTCGGGTGTTACAAACTCTCATGGTGTGACGGGCGGTGTGTACAAGGCCCGGGAACGTATTCACCGCGGCGTGCTGATCCGCGATTACTAGCGATTCCGACTTCGTGTAGGCGAGTTGCAGCCTACAGTCCGAACTGAGAATGGCTTTAAGAGATTAGCTTGACCTCGCGGTCTCGCAACTCGTTGTACCATCCATTGTAGCACGTGTGTAGCCCAGGTCATAAGGGGCATGATGATTTGACGTCATCCCCACCTTCCTCCGGTTTGTCACCGGCAGTCTTACTAGAGTGCCCAACTAAATGCTGGCAACTAGTCATAAGGGTTGCGCTCGTTGCGGGACTTAACCCAACATCTCACGACACGAGCTGACGACAACCATGCACCACCTGTCATTTTGCCCCCGAAGGGGAAACCTGATCTCTCAGGTGATCAAAAGATGTCAAGACCTGGTAAGGTTCTTCGCGTTGCTTCGAATTAAACCACATGCTCCACCGCTTGTGCGGGCCCCCGTCAATTCCTTTGAGTTTCAACCTTGCGGTCGTACTCCCCAGGCGGAATGCTTAATGCGTTAGCTGCGGCACTGAAGGGCGGAAACCCTCCAACACCTAGCATTCATCGTTTACGGCATGGACTACCAGGGTATCTAATCCTGTTCGCTACCCATGCTTTCGAGCCTCAGCGTCAGTTACAGACCAGACAGCCGCCTTCGCCACTGGTGTTCTTCCATATATCTACGCATTTCACCGCTACACATGGAGTTCCACTGTCCTCTTCTGCACTCAAGTTTCCCAGTTTCCGATGCGCTTCCTCGGTTAAGCCGAGGGCTTTCACATCAGACTTAAAAAACCGCCTGCGCTCGCTTTACGCCCAATAAATCCGGATAACGCTTGCCACCTACGTATTACCGCGGCTGCTGGCACGTAGTTAGCCGTGGCTTTCTGGTTGGATACCGTCACGCCGACAACAGTTACTCTGCCGACCATTCTTCTCCAACAACAGAGTTTTACGACCCGAAAGCCTTCTTCACTCACGCGGCGTTGCTCCATCAGACTTGCGTCCATTGTGGAAGATTCCCTACTGCTGCCTCCCGTAGGAGTTTGGGCCGTGTCTCAGTCCCAATGTGGCCGATCAACCTCTCAGTTCGGCTACGTATCATCGCCTTGGTGAGCCATTACCTCACCAACTAGCTAATACGCCGCGGGTCCATCCAAAAGCGATAGCTTACGCCATCTTTCAGCCAAGAACCATGCGGTTCTTGGATCTATGCGGTATTAGCATCTGTTTCCAAATGTTATCCCCCACTTAAGGGCAGGTTACCCACGTGTTACTCACCCGTCCGCCACTCGTTCCATGTTGAATCTCGGTGCAAGCACCGATCATCAACGAGAACTCGTCGACTGCATGTATAGACCCG

>Lactobacillus plantarum  APW2112A
CTGTCACTTAGGCGGCTGGTTCCTAAAGGTTACCCCACCGACTTTGGGTGTTACAAACTCTCATGGTGTGACGGGCGGTGTGTACAAGGCCCGGGAACGTATTCACCGCGGCATGCTGATCCGCGATTACTAGCGATTCCGACTTCATGTAGGCGAGTTGCAGCCTACAATCCGAACTGAGAATGGCTTTAAGAGATTAGCTTACTCTCGCGAGTTCGCAACTCGTTGTACCATCCATTGTAGCACGTGTGTAGCCCAGGTCATAAGGGGCATGATGATTTGACGTCATCCCCACCTTCCTCCGGTTTGTCACCGGCAGTCTCACCAGAGTGCCCAACTTAATGCTGGCAACTGATAATAAGGGTTGCGCTCGTTGCGGGACTTAACCCAACATCTCACGACACGAGCTGACGACAACCATGCACCACCTGTATCCATGTCCCCGAAGGGAACGTCTAATCTCTTAGATTTGCATAGTATGTCAAGACCTGGTAAGGTTCTTCGCGTAGCTTCGAATTAAACCACATGCTCCACCGCTTGTGCGGGCCCCCGTCAATTCCTTTGAGTTTCAGCCTTGCGGCCGTACTCCCCAGGCGGAATGCTTAATGCGTTAGCTGCAGCACTGAAGGGCGGAAACCCTCCAACACTTAGCATTCATCGTTTACGGTATGGACTACCAGGGTATCTAATCCTGTTTGCTACCCATACTTTCGAGCCTCAGCGTCAGTTACAGACCAGACAGCCGCCTTCGCCACTGGTGTTCTTCCATATATCTACGCATTTCACCGCTACACATGGAGTTCCACTGTCCTCTTCTGCACTCAAGTTTCCCAGTTTCCGATGCACTTCTTCGGTTGAGCCGAAGGCTTTCACATCAGACTTAAAAAACCGCCTGCGCTCGCTTTACGCCCAATAAATCCGGACAACGCTTGCCACCTACGTATTACCGCGGCTGCTGGCACGTAGTTAGCCGTGGCTTTCTGGTTAAATACCGTCAATACCTGAACAGTTACTCTTAGATATGTTCTTCTTTAACAACAGAGTTTTACGAGCCGAAACCCTTCTTCACTCACGGGGCGTTGCTCCATCAGACTTTCGTCCATTGTGGAAGATTCCCTACTGCTGCCTCCCGTAGGAGTTTGGGCCGTGTCTCAGTCCCAATGTGGCCGATTACCCTCTCAGGTCGGCTACGTATCATTGCCATGGTGAGCCGTTACCTCACCATCTAGCTAATACGCCGCGGGACCATCCAAAAGTGATAGCCGAAGCCATCTTTCAAACTCGGACCATGCGGTCCAAGTTGTTATGCGGTATTAGCATCTGTTTCCAGGTGTTATCCCCCGCTTCTGGGCAGGTTTCCCACGTGTTACTCACCAGTTCGCCACTCACTCAAATGTAAATCATGATGCAAGCACCAATCAATACCAGAGTTCTTCGACTGCAGTTAGCACGCCGCAC

>Lactobacillus brevis  APW2112B
ATGTCAGCATGAGACTAGCTCGTATAGCCGTAAGTGCTTATCTTCTAAGCGGCTTTGGGTGTTACAAACTCTCATGGTGTGACGGGCGGTGTGTACAAGGCCCGGGAACGTATTCACCGCGGCATGCTGATCCGCGATTACTAGCGATTCCAACTTCATGTAGGCGAGTTGCAGCCTACAATCCGAACTGAGAACGGCTTTAAGAGATTAGCTTAGCCTCACGACTTCGCAACTCGTTGTACCGTCCATTGTAGCACGTGTGTAGCCCAGGTCATAAGGGGCATGATGATTTGACGTCATCCCCACCTTCCTCCGGTTTGTCACCGGCAGTCTCACCAGAGTGCCCAACTGAATGCTGGCAACTGATAATAAGGGTTGCGCTCGTTGCGGGACTTAACCCAACATCTCACGACACGAGCTGACGACAACCATGCACCACCTGTCATTCTGTCCCCGAAGGGAACGTCTTATCTCTAAGATTGGCAGAAGATGTCAAGACCTGGTAAGGTTCTTCGCGTAGCTTCGAATTAAACCACATGCTCCACCGCTTGTGCGGGCCCCCGTCAATTCCTTTGAGTTTCAACCTTGCGGTCGTACTCCCCAGGCGGAGTGCTTAATGCGTTAGCTGCAGCACTGAAGGGCGGAAACCCTCCAACACTTAGCACTCATCGTTTACGGCATGGACTACCAGGGTATCTAATCCTGTTCGCTACCCATGCTTTCGAGCCTCAGCGTCAGTTACAGACTAGACAGCCGCCTTCGCCACTGGTGTTCTTCCATATATCTACGCATTCCACCGCTACACATGGAGTTCCACTGTCCTCTTCTGCACTCAAGTCTCCCAGTTTCCGATGCACTTCTCCGGTTAAGCCGAAGGCTTTCACATCAGACTTAAAAAACCGCCTGCGCTCGCTTTACGCCCAATAAATCCGGACAACGCTTGCCACCTACGTATTACCGCGGCTGCTGGCACGTAGTTAGCCGTGGCTTTCTGGTTAAATACCGTCAACCCTTGAACAGTTACTCTCAAAGGTGTTCTTCTTTAACAACAGAGTTTTACGAGCCGAAACCCTTCTTCACTCACGCGGCATTGCTCCATCAGACTTTCGTCCATTGTGGAAGATTCCCTACTGCTGCCTCCCGTAGGAGTTTGGGCCGTGTCTCAGTCCCAATGTGGCCGATTACCCTCTCAGGTCGGCTACGTATCATCGTCTTGGTGGGCCTTTACCTCACCAACTAAGTAATACGCCGCGGGATCATCCAGAAGTGATAGCCGAAGCCACCTTTCAAACAAAATCCCTGCGGATTTTGTTGTTATACGGTATTAGCACCTGTTTCCAAGTGTTATCCCCTGCTTCTGGGCAGATTTCCCACGTGTTACTCACCAGTTCGCCACTCGCTTCATTGTTGAAATCAGTGCAAGCACGTCATTCACACGGAACGCTCGTACGACTGCATGTATATACAGCTGCAC

>Lactobacillus casei APW2112D
GTCACTTAGACGGCTCGCTCCCTAAAGGGTTACGCCACCGGCTTCGGGTGTTACAAACTCTCATGGTGTGACGGGCGGTGTGTACAAGGCCCGGGAACGTATTCACCGCGGCGTGCTGATCCGCGATTACTAGCGATTCCGACTTCGTGTAGGCGAGTTGCAGCCTACAGTCCGAACTGAGAATGGCTTTAAGAGATTAGCTTGACCTCGCGGTCTCGCAACTCGTTGTACCATCCATTGTAGCACGTGTGTAGCCCAGGTCATAAGGGGCATGATGATTTGACGTCATCCCCACCTTCCTCCGGTTTGTCACCGGCAGTCTTACTAGAGTGCCCAACTAAATGCTGGCAACTAGTCATAAGGGTTGCGCTCGTTGCGGGACTTAACCCAACATCTCACGACACGAGCTGACGACAACCATGCACCACCTGTCATTTTGCCCCCGAAGGGGAAACCTGATCTCTCAGGTGATCAAAAGATGTCAAGACCTGGTAAGGTTCTTCGCGTTGCTTCGAATTAAACCACATGCTCCACCGCTTGTGCGGGCCCCCGTCAATTCCTTTGAGTTTCAACCTTGCGGTCGTACTCCCCAGGCGGAATGCTTAATGCGTTAGCTGCGGCACTGAAGGGCGGAAACCCTCCAACACCTAGCATTCATCGTTTACGGCATGGACTACCAGGGTATCTAATCCTGTTCGCTACCCATGCTTTCGAGCCTCAGCGTCAGTTACAGACCAGACAGCCGCCTTCGCCACTGGTGTTCTTCCATATATCTACGCATTTCACCGCTACACATGGAGTTCCACTGTCCTCTTCTGCACTCAAGTTTCCCAGTTTCCGATGCGCTTCCTCGGTTAAGCCGAGGGCTTTCACATCAGACTTAAAAAACCGCCTGCGCTCGCTTTACGCCCAATAAATCCGGATAACGCTTGCCACCTACGTATTACCGCGGCTGCTGGCACGTAGTTAGCCGTGGCTTTCTGGTTGGATACCGTCACGCCGACAACAGTTACTCTGCCGACCATTCTTCTCCAACAACAGAGTTTTACGACCCGAAAGCCTTCTTCACTCACGGGGCGTTGCTCCATCAGACTTGCGTCCATTGTGGAAGATTCCCTACTGCTGCCTCCCGTAGGAGTTTGGGCCGTGTCTCAGTCCCAATGTGGCCGATCAACCTCTCAGTTCGGCTACGTATCATCGCCTTGGTGAGCCATTACCTCACCAACTAGCTAATACGCCGCGGGTCCATCCAAAAGCGATAGCTTACGCCATCTTTCAGCCAAGAACCATGCGGTTCTTGGATCTATGCGGTATTAGCATCTGTTTCCAAATGTTATCCCCCACTTAAGGGCAGGTTACCCACGTGTTACTCACCCGTCCGCCACTCGTTCCATGTTGAATCTCGGTGCAAGCACCGATCATCAACGAGAACTCGTTCGACTGCATGTATAGGAGCG

>Lactobacillus brevis  APW2112C
ACCATTCTGTCACTTAGACGGCTGACTCCCGAAGGTTATCTCACCGGCTTTGGGTGTTACAAACTCTCATGGTGTGACGGGCGGTGTGTACAAGGCCCGGGAACGTATTCACCGCGGCATGCTGATCCGCGATTACTAGCGATTCCAACTTCATGTAGGCGAGTTGCAGCCTACAATCCGAACTGAGAACGGCTTTAAGAGATTAGCTTAGCCTCACGACTTCGCAACTCGTTGTACCGTCCATTGTAGCACGTGTGTAGCCCAGGTCATAAGGGGCATGATGATTTGACGTCATCCCCACCTTCCTCCGGTTTGTCACCGGCAGTCTCACCAGAGTGCCCAACTGAATGCTGGCAACTGATAATAAGGGTTGCGCTCGTTGCGGGACTTAACCCAACATCTCACGACACGAGCTGACGACAACCATGCACCACCTGTCATTCTGTCCCCGAAGGGAACGTCTTATCTCTAAGATTGGCAGAAGATGTCAAGACCTGGTAAGGTTCTTCGCGTAGCTTCGAATTAAACCACATGCTCCACCGCTTGTGCGGGCCCCCGTCAATTCCTTTGAGTTTCAACCTTGCGGTCGTACTCCCCAGGCGGAGTGCTTAATGCGTTAGCTGCAGCACTGAAGGGCGGAAACCCTCCAACACTTAGCACTCATCGTTTACGGCATGGACTACCAGGGTATCTAATCCTGTTCGCTACCCATGCTTTCGAGCCTCAGCGTCAGTTACAGACTAGACAGCCGCCTTCGCCACTGGTGTTCTTCCATATATCTACGCATTCCACCGCTACACATGGAGTTCCACTGTCCTCTTCTGCACTCAAGTCTCCCAGTTTCCGATGCACTTCTCCGGTTAAGCCGAAGGCTTTCACATCAGACTTAAAAAACCGCCTGCGCTCGCTTTACGCCCAATAAATCCGGACAACGCTTGCCACCTACGTATTACCGCGGCTGCTGGCACGTAGTTAGCCGTGGCTTTCTGGTTAAATACCGTCAACCCTTGAACAGTTACTCTCAAAGGTGTTCTTCTTTAACAACAGAGTTTTACGAGCCGAAACCCTTCTTCACTCACGCGGCATTGCTCCATCAGACTTTCGTCCATTGTGGAAGATTCCCTACTGCTGCCTCCCGTAGGAGTTTGGGCCGTGTCTCAGTCCCAATGTGGCCGATTACCCTCTCAGGTGGGCTACGTATAATCGTCTTGGTGGGCCTTTACCTCACCAACTAACTATTACGCCGCGGGATCATCCAGAAGTGATAGCCGAAGCCACCTTTCAAACAAAATCCCTGCGGATTTTGTTGTTATACGGTATTAGCACCTGTTTCCAAGTGTTATCCCCTGCTTCTGGGCAGATTTCCCACGTGTTACTCACCACTTCGCCACTCGCTTCATTGTTAAAATCAGTGCAAGCACGTCATTCAACGGAAGCTCGTTCGACTGCATGTATAGGCTGCGCA

>Lactobacillus fermentum APW1317
GTCACCTTAGGCGGCTGGCTCCTAAAGGTTACCCCACCGACTTTGGGTGTTACAAACTCTCATGGTGTGACGGGCGGTGTGTACAAGGCCCGGGAACGTATTCACCGCGGCATGCTGATCCGCGATTACTAGCGATTCCGACTTCGTGCAGGCGAGTTGCAGCCTGCAGTCCGAACTGAGAACGGTTTTAAGAGATTTGCTTGCCCTCGCGAGTTCGCGACTCGTTGTACCGTCCATTGTAGCACGTGTGTAGCCCAGGTCATAAGGGGCATGATGATCTGACGTCGTCCCCACCTTCCTCCGGTTTGTCACCGGCAGTCTCACTAGAGTGCCCAACTTAATGCTGGCAACTAGTAACAAGGGTTGCGCTCGTTGCGGGACTTAACCCAACATCTCACGACACGAGCTGACGACGACCATGCACCACCTGTCATTGCGTTCCCGAAGGAAACGCCCTATCTCTAGGGTTGGCGCAAGATGTCAAGACCTGGTAAGGTTCTTCGCGTAGCTTCGAATTAAACCACATGCTCCACCGCTTGTGCGGGCCCCCGTCAATTCCTTTGAGTTTCAACCTTGCGGTCGTACTCCCCAGGCGGAGTGCTTAATGCGTTAGCTCCGGCACTGAAGGGCGGAAACCCTCCAACACCTAGCACTCATCGTTTACGGCATGGACTACCAGGGTATCTAATCCTGTTCGCTACCCATGCTTTCGAGTCTCAGCGTCAGTTGCAGACCAGGTAGCCGCCTTCGCCACTGGTGTTCTTCCATATATCTACGCATTCCACCGCTACACATGGAGTTCCACTACCCTCTTCTGCACTCAAGTTATCCAGTTTCCGATGCACTTCTCCGGTTAAGCCGAAGGCTTTCACATCAGACTTAGAAAACCGCCTGCACTCTCTTTACGCCCAATAAATCCGGATAACGCTTGCCACCTACGTATTACCGCGGCTGCTGGCACGTAGTTAGCCGTGACTTTCTGGTTAAATACCGTCAACGTATGAACAGTTACTCTCATACGTGTTCTTCTTTAACAACAGAGCTTTACGAGCCGAAACCCTTCTTCACTCACGCGGTGTTGCTCCATCAGGCTTGCGCCCATTGTGGAAGATTCCCTACTGCTGCCTCCCGTAGGAGTATGGGCCGTGTCTCAGTCCCATTGTGGCCGATCAGTCTCTCAACTTGGCTATGCATCATCGCCTTGGTAGGCCGTTACCCCACCAACAAGCTAATGCACCGCAGGTCCATCCAGAAGTGATAGCGAGAAGCCATCTTTTAAGCGTTGTTCATGCGAACAACGTTGTTATGCGGTATTAGCATCTGTTTCCAAATGTTGTCCCCCGCTTCTGGGCAGGTTACCTACGTGTTACTCACCCGTCCGCCACTCGTTGGCGACCAAAATCAATCAGGTGCAAGCACCATCAATCAATTGGGCCAACGCGTTCGACTGCATGTATAGGCACCGC

>Lactobacillus fermentum APW1434E
TGTCACTTAGGCGGCTGGCTCCTAAAGGTTACCCCACCGACTTTGGGTGTTACAAACTCTCATGGTGTGACGGGCGGTGTGTACAAGGCCCGGGAACGTATTCACCGCGGCATGCTGATCCGCGATTACTAGCGATTCCGACTTCGTGCAGGCGAGTTGCAGCCTGCAGTCCGAACTGAGAACGGTTTTAAGAGATTTGCTTGCCCTCGCGAGTTCGCGACTCGTTGTACCGTCCATTGTAGCACGTGTGTAGCCCAGGTCATAAGGGGCATGATGATCTGACGTCGTCCCCACCTTCCTCCGGTTTGTCACCGGCAGTCTCACTAGAGTGCCCAACTTAATGCTGGCAACTAGTAACAAGGGTTGCGCTCGTTGCGGGACTTAACCCAACATCTCACGACACGAGCTGACGACGACCATGCACCACCTGTCATTGCGTTCCCGAAGGAAACGCCCTATCTCTAGGGTTGGCRCAAGATGTCAAGACCTGGTAAGGTTCTTCGCGTAGCTTCGAATTAAACCACATGCTCCACCGCTTGTGCGGGCCCCCGTCAATTCCTTTGAGTTTCAACCTTGCGGTCGTACTCCCCAGGCGGAGTGCTTAATGCGTTAGCTCCGGCACTGAAGGGCGGAAACCCTCCAACACCTAGCACTCATCGTTTACGGCATGGACTACCAGGGTATCTAATCCTGTTCGCTACCCATGCTTTCGAGTCTCAGCGTCAGTTGCAGACCAGGTAGCCGCCTTCGCCACTGGTGTTCTTCCATATATCTACGCATTCCACCGCTACACATGGAGTTCCACTACCCTCTTCTGCACTCAAGTTATCCAGTTTCCGATGCACTTCTCCGGTTAAGCCGAAGGCTTTCACATCAGACTTAGAAAACCGCCTGCACTCTCTTTACGCCCAATAAATCCGGATAACGCTTGCCACCTACGTATTACCGCGGCTGCTGGCACGTAGTTAGCCGTGACTTTCTGGTTAAATACCGTCAACGTATGAACAGTTACTCTCATACGTGTTCTTCTTTAACAACAGAGCTTTACGAGCCGAAACCCTTCTTCACTCACGCGGTGTTGCTCCATCAGGCTTGCGCCCATTGTGGAAGATTCCCTACTGCTGCCTCCCGTAGGAGTATGGGCCGTGTCTCAGTCCCATTGTGGCCGATCAGTCTCTCAACTCGGCTATGCATCATCGCCTTGGTAGGCCGTTACCCCACCAACAAGCTAATGCACCGCAGGTCCATCCAGAAGTGATAGCGAGAAGCCATCTTTTAAGCGTTGTTCATGCGAACAACGCTGTTATGCGGTATTAGCATCTGTTTCCAAATGTTGTCCCCCGCTTCTGGGCAGGTTACCTACGTGTTACTCACCCGTCCGCCACTCGTTGGCGACCAAAATCAATCAGGTGCAAGCACCATCAATCAATTTGGGCCAACGCGTCGACTGCATGTATAG

>Lactobacillus casei APW1434D
TGTCACTTAGACGGCTCGCTCCCTAAAGGGTTACGCCACCGGCTTCGGGTGTTACAAACTCTCATGGTGTGACGGGCGGTGTGTACAAGGCCCGGGAACGTATTCACCGCGGCGTGCTGATCCGCGATTACTAGCGATTCCGACTTCGTGTAGGCGAGTTGCAGCCTACAGTCCGAACTGAGAATGGCTTTAAGAGATTAGCTTGACCTCGCGGTCTCGCAACTCGTTGTACCATCCATTGTAGCACGTGTGTAGCCCAGGTCATAAGGGGCATGATGATTTGACGTCATCCCCACCTTCCTCCGGTTTGTCACCGGCAGTCTTACTAGAGTGCCCAACTAAATGCTGGCAACTAGTCATAAGGGTTGCGCTCGTTGCGGGACTTAACCCAACATCTCACGACACGAGCTGACGACAACCATGCACCACCTGTCATTTTGCCCCCGAAGGGGAAACCTGATCTCTCAGGTGATCAAAAGATGTCAAGACCTGGTAAGGTTCTTCGCGTTGCTTCGAATTAAACCACATGCTCCACCGCTTGTGCGGGCCCCCGTCAATTCCTTTGAGTTTCAACCTTGCGGTCGTACTCCCCAGGCGGAATGCTTAATGCGTTAGCTGCGGCACTGAAGGGCGGAAACCCTCCAACACCTAGCATTCATCGTTTACGGCATGGACTACCAGGGTATCTAATCCTGTTCGCTACCCATGCTTTCGAGCCTCAGCGTCAGTTACAGACCAGACAGCCGCCTTCGCCACTGGTGTTCTTCCATATATCTACGCATTTCACCGCTACACATGGAGTTCCACTGTCCTCTTCTGCACTCAAGTTTCCCAGTTTCCGATGCGCTTCCTCGGTTAAGCCGAGGGCTTTCACATCAGACTTAAAAAACCGCCTGCGCTCGCTTTACGCCCAATAAATCCGGATAACGCTTGCCACCTACGTATTACCGCGGCTGCTGGCACGTAGTTAGCCGTGGCTTTCTGGTTGGATACCGTCACGCCGACAACAGTTACTCTGCCGACCATTCTTCTCCAACAACAGAGTTTTACGACCCGAAAGCCTTCTTCACTCACGCGGCGTTGCTCCATCAGACTTGCGTCCATTGTGGAAGATTCCCTACTGCTGCCTCCCGTAGGAGTTTGGGCCGTGTCTCAGTCCCAATGTGGCCGATCAACCTCTCAGTTTGGCTACGTATCATCGCCTTGGTGAGCCATTACCTCACCAACTAGCTAATACGCCGCGGGTCCATCCAAAAGCGATAGCTTACGCCATCTTTCAGCCAAGAACCATGCGGTTCTTGGATCTATGCGGTATTAGCATCTGTTTCCAAATGTTATCCCCCACTTAAGGGCAGGTTACCCACGTGTTACTCACCCGTCCGCCACTCGTTCCATGTTGAATCTCGGTGCAAGCACCGATCATCAACGAGAACTCGTTCGACTGCATGTATAGCACGCC


>Lactobacillus casei APW1161A
CACTTAGACGGCTCGCTCCCTAAAGGGTTACGCCACCGGCTTCGGGTGTTACAAACTCTCATGGTGTGACGGGCGGTGTGTACAAGGCCCGGGAACGTATTCACCGCGGCGTGCTGATCCGCGATTACTAGCGATTCCGACTTCGTGTAGGCGAGTTGCAGCCTACAGTCCGAACTGAGAATGGCTTTAAGAGATTAGCTTGACCTCGCGGTCTCGCAACTCGTTGTACCATCCATTGTAGCACGTGTGTAGCCCAGGTCATAAGGGGCATGATGATTTGACGTCATCCCCACCTTCCTCCGGTTTGTCACCGGCAGTCTTACTAGAGTGCCCAACTAAATGCTGGCAACTAGTCATAAGGGTTGCGCTCGTTGCGGGACTTAACCCAACATCTCACGACACGAGCTGACGACAACCATGCACCACCTGTCATTTTGCCCCCGAAGGGGAAACCTGATCTCTCAGGTGATCAAAAGATGTCAAGACCTGGTAAGGTTCTTCGCGTTGCTTCGAATTAAACCACATGCTCCACCGCTTGTGCGGGCCCCCGTCAATTCCTTTGAGTTTCAACCTTGCGGTCGTACTCCCCAGGCGGAATGCTTAATGCGTTAGCTGCGGCACTGAAGGGCGGAAACCCTCCAACACCTAGCATTCATCGTTTACGGCATGGACTACCAGGGTATCTAATCCTGTTCGCTACCCATGCTTTCGAGCCTCAGCGTCAGTTACAGACCAGACAGCCGCCTTCGCCACTGGTGTTCTTCCATATATCTACCGCATTTCACCGCTACACATGGGAGTTCCACTTGTCCTCTTCTGGCACTCAAGTTTCCCAGTTTCCCGATGCGCTTCCTCCGGTTAAGCCGAGGGCTTTCACATCAGACTTAAAAAACCGCCTGCGCTCGCTTTACGCCCAATAAATCCGGATAACGCTTGCCACCTACGTATTACCGCGGCTGCTGGCACGTAGTTAGCCGTGGCTTTCTGGTTGGATACCGTCACGCCGACAACAGTTACTCTGCCGACCATTCTTCTCCAACAACAGAGTTTTACGACCCGAAAGCCTTCTTCACTCACGCGGCGTTGCTCCATCAGACTTGCGTCCATTGTGGAAGATTCCCTACTGCTGCCTCCCGTAGGAGTTTGGGCCGTGTCTCAGTCCCAATGTGGCCGATCAACCTCTCAGTTCGGCTACGTATCATCGCCTTGGTGAGCCATTACCTCACCAACTAGCTAATACGCCGCGGGTCCATCCAAAAGCGATAGCTTACGCCATCTTTCAGCCAAGAACCATGCGGTTCTTGGATCTATGCGGTATTAGCATCTGTTTCCAAATGTTATCCCCCACTTAAGGGCAGGTTACCCACGTGTTACTCACCCGTCCGCCACTCGTTCCATGTTGAATCTCGGTGCAAGCACCAATCATCAACGAGAACTCGTCGACTGCATGTATAGCCGCCG

>Lactobacillus casei APW2439C
CCACCTTAGACGGCTCGCTCCCTAAAGGGTTACGCCACCGGCTTCGGGTGTTACAAACTCTCATGGTGTGACGGGCGGTGTGTACAAGGCCCGGGAACGTATTCACCGCGGCGTGCTGATCCGCGATTACTAGCGATTCCGACTTCGTGTAGGCGAGTTGCAGCCTACAGTCCGAACTGAGAATGGCTTTAAGAGATTAGCTTGACCTCGCGGTCTCGCAACTCGTTGTACCATCCATTGTAGCACGTGTGTAGCCCAGGTCATAAGGGGCATGATGATTTGACGTCATCCCCACCTTCCTCCGGTTTGTCACCGGCAGTCTTACTAGAGTGCCCAACTAAATGCTGGCAACTAGTCATAAGGGTTGCGCTCGTTGCGGGACTTAACCCAACATCTCACGACACGAGCTGACGACAACCATGCACCACCTGTCATTTTGCCCCCGAAGGGGAAACCTGATCTCTCAGGTGATCAAAAGATGTCAAGACCTGGTAAGGTTCTTCGCGTTGCTTCGAATTAAACCACATGCTCCACCGCTTGTGCGGGCCCCCGTCAATTCCTTTGAGTTTCAACCTTGCGGTCGTACTCCCCAGGCGGAATGCTTAATGCGTTAGCTGCGGCACTGAAAGGCGGAAACCCTCCAACACCTAGCATTCATCGTTTACGGCATGGACTACCAGGGTATCTAATCCTGTTCGCTACCCATGCTTTCGAGCCTCAGCGTCAGTTACAGACCAGACAGCCGCCTTCGCCACTGGTGTTCTTCCATATATCTACGCATTTCACCGCTACACATGGAGTTCCACTGTCCTCTTCTGCACTCAAGTTTCCCAGTTTCCGATGCGCTTCCTCGGTTAAGCCGAGGGCTTTCACATCAGACTTAAAAAACCGCCTGCGCTCGCTTTACGCCCAATAAATCCCGGATAACGCTTGCCACCTACGTATTACCGCGGCTGCTGGCACGTAGTTAGCCGTGGCTTTCTGGTTGGATACCGTCACGCCGACAACAGTTACTCTGCCGACCATTCTTCTCCAACAACAGAGTTTTACGACCCGAAAGCCTTCTTCACTCACGCGGCGTTGCTCCATCAGACTTGCGTCCATTGTGGAAGATTCCCTACTGCTGCCTCCCGTAGGAGTTTGGGCCGTGTCTCAGTCCCAATGTGGCCGATCAACCTCTCAGTTCGGCTACGTATCACGCCTTGGTGAGCCATTACCTCACCAACTAGCTAATACGCCGCGGGTCCATCCAAAAGCGATAGCTTACGCCATCTTTCAGCCAAGAACCATGCGGTTCTTGGATCTATGCGGTATTAGCATCTGTTTCCAAATGTTATCCCCCACTTAAGGGCAGGTTACCCACGTGTTACTCACCCGTCCGCCACTCGTTCCATGTTGAATCTCGGTGCAAGCACCGATCATCAACGAGAACTCGTTCGACTGCATGTATAGAC

>Lactobacillus casei APW2439B
GGTCACTTAGACGGCTCGCTCCCTAAAGGGTTACGCCACCGGCTTCGGGTGTTACAAACTCTCATGGTGTGACGGGCGGTGTGTACAAGGCCCGGGAACGTATTCACCGCGGCGTGCTGATCCGCGATTACTAGCGATTCCGACTTCGTGTAGGCGAGTTGCAGCCTACAGTCCGAACTGAGAATGGCTTTAAGAGATTAGCTTGACCTCGCGGTCTCGCAACTCGTTGTACCATCCATTGTAGCACGTGTGTAGCCCAGGTCATAAGGGGCATGATGATTTGACGTCATCCCCACCTTCCTCCGGTTTGTCACCGGCAGTCTTACTAGAGTGCCCAACTAAATGCTGGCAACTAGTCATAAGGGTTGCGCTCGTTGCGGGACTTAACCCAACATCTCACGACACGAGCTGACGACAACCATGCACCACCTGTCATTTTGCCCCCAAAGGGGAAACCTGATCTCTCAGGTGATCAAAAGATGTCAAGACCTGGTAAGGTTCTTCGCGTTGCTTCGAATTAAACCACATGCTCCACCGCTTGTGCGGGCCCCCGTCAATTCCTTTGAGTTTCAACCTTGCGGTCGTACTCCCCAGGCGGAATGCTTAATGCGTTAGCTGCGGCACTGAAAGGCGGAAACCCTCCAACACCTAGCATTCATCGTTTACGGCATGGACTACCAGGGTATCTAATCATGTTCGCTACCCATGCTGTCGAGCCTCAGCGTCAGTTACAGACCAGACAGCCGCCTTCGGCACTGGTGTTCTTCCATATATCTACGCATTTCACCGCTACACATGGAGTWYCACTGTCCTCTTCTGCACTCAAGTTTCCCAGTTTCCGATGCGCTTCCTCGGTTAAGCCGAGGGCTTTCACATCAGACTTAAAAAAACCGCCTGCGCTCGCTTTACGCCCAATAAATCCGGATAACGCTTGCCACCTACGTATTACCGCGGCTGCTGGCACGTAGTTAGCCGTGGCTTTCTGGTTGGATACCGTCACGCCGACAACAGTTACTCTGCCGACMATTCTTCTCCAACAACAGAGTTTTACGACCCGAAAGCCTTCTTCACTCACGCGGCGTTGCTCCATCAGACTTGCGTCCATTGTGGAAGATTCCCTACTGCTGCCTCCCGTAGGAGTTTGGGCCGTGTCTCAGTCCCAATGTGGCCGATCAACCTCTCAGTTCGGCTACGTATCATCGCCTTGGTGAGCCATTACCTCACCAACTAGCTAATACGCCGCGGGTCCATCCAAAAGCGATAGCTTACGCCATCTTTCAGCCAAGAACCATGCGGTTCTTGGATCTATGCGGTATTAGCATCTGTTTCCAAATGTTATCCCCCACTTAAGGGCAGGTTACCCACGTGTTACTCACCCGTCCGCCACTCGTTCCATGTTGAATCTCGGTGCAAGCACCGATCATCAACGAGAACTCGTTCGACTGCATGTATAGCA

>Lactobacillus casei APW2439A
GAGTCCTATACATGCAGTCGAACGAGTTCCTCCGTTGATGATCGGTGCTTGCACCGAGATTCAACATGGAACGAGTGGCGGACGGGTGAGTAACACGTGGGTAACCTGCCCTTAAGTGGGGGATAACATTTGGAAACAGATGCTAATACCGCATAGATCCAAGAACCGCATGGTTCTTGGCTGAAAGATGGCGTAAGCTATCGCTTTTGGATGGACCCGCGGCGTATTAGCTAGTTGGTGAGGTAATGGCTCACCAAGGCGATGATACGTAGCCGAACTGAGAGGTTGATCGGCCACATTGGGACTGAGACACGGCCCAAACTCCTACGGGAGGCAGCAGTAGGGAATCTTCCACAATGGACGCAAGTCTGATGGAGCAACGCCGCGTGAGTGAAGAAGGTTTCGGTCGAAACTCTGTTGTGGAGAAGATGTCGGAGATACTGTGCGCGTGACGTTCCACCAAAAGCACGGTAACTACTGCACACCCGTATACTAGTGGAACGTACCGGTTTTTGGCTAACACGAGGTTTTAATCTGTGGAACCTCGCTACCAGACGACGAATGGAACTGTGAAAGCATGACTCTGGACGTGATGTAATTGAACCATGCAAGGTTTGCGATAGTAGTCAACTGGACAAGTAACCGATCTGCTACAATCAGTGAGGTCCCTCTCCCACTAGTCCGGTCACCAGAGATCAGTATGGCCAACGACGGTATCACCCGACGCATGTGTTCAAAAGCCTCGGACATTGGATTTCACGGTGACGCCACTCTGGATAGGGA

>Lactobacillus casei APW1161B
GTCACTTAGACGGCTCGCTCCCTAAAGGGTTACGCCACCGGCTTCGGGTGTTACAAACTCTCATGGTGTGACGGGCGGTGTGTACAAGGCCCGGGAACGTATTCACCGCGGCGTGCTGATCCGCGATTACTAGCGATTCCGACTTCGTGTAGGCGAGTTGCAGCCTACAGTCCGAACTGAGAATGGCTTTAAGAGATTAGCTTGACCTCGCGGTCTCGCAACTCGTTGTACCATCCATTGTAGCACGTGTGTAGCCCAGGTCATAAGGGGCATGATGATTTGACGTCATCCCCACCTTCCTCCGGTTTGTCACCGGCAGTCTTACTAGAGTGCCCAACTAAATGCTGGCAACTAGTCATAAGGGTTGCGCTCGTTGCGGGACTTAACCCAACATCTCACGACACGAGCTGACGACAACCATGCACCACCTGTCATTTTGCCCCCGAAGGGGAAACCTGATCTCTCAGGTGATCAAAAGATGTCAAGACCTGGTAAGGTTCTTCGCGTTGCTTCGAATTAAACCACATGCTCCACCGCTTGTGCGGGCCCCCGTCAATTCCTTTGAGTTTCAACCTTGCGGTCGTACTCCCCAGGCGGAATGCTTAATGCGTTAGCTGCGGCACTGAAGGGCGGAAACCCTCCAACACCTAGCATTCATCGTTTACGGCATGGACTACCAGGGTATCTAATCCTGTTCGCTACCCATGCTTTCGAGCCTCAGCGTCAGTTACAGACCAGACAGCCGCCTTCGCCACTGGTGTTCTTCCATATATCTACGCATTTCACCGCTACACATGGAGTTCCACTGTCCTCTTCTGCACTCAAGTTTCCCAGTTTCCGATGCGCTTCCTCGGTTAAGCCGAGGGCTTTCACATCAGACTTAAAAAACCGCCTGCGCTCGCTTTACGCCCAATAAATCCGGATAACGCTTGCCACCTACGTATTACCGCGGCTGCTGGCACGTAGTTAGCCGTGGCTTTCTGGTTGGATACCGTCACGCCGACAACAGTTACTCTGCCGACCATTCTTCTCCAACAACAGAGTTTTACGACCCGAAAGCCTTCTTCACTCACGCGGCGTTGCTCCATCAGACTTGCGTCCATTGTGGAAGATTCCCTACTGCTGCCTCCCGTAGGAGTTTGGGCCGTGTCTCAGTCCCAATGTGGCCGATCAACCTCTCAGTTCGGCTACGTATCATCGCCTTGGTGAGCCATTACCTCACCAACTAGCTAATACGCCGCGGGTCCATCCAAAAGCGATAGCTTACGCCATCTTTCAGCCAAGAACCATGCGGTTCTTGGATCTATGCGGTATTAGCATCTGTTTCCAAATGTTATCCCCCACTTAAGGGCAGGTTACCCACGTGTTACTCACCCGTCCGCCACTCGTTCCATGTTGAATCTCGGTGCAAGCACCGATCATCAACGAGGAACTCGTTCGGACTGCATGTATAGGCACGCC

>Lactobacillus pantheris APW1161C
CTGTCACTTAGACGGCTAGCTCCCTGAAGGGTTGCCCCACCGGCTTTGGGTGTTACAAACTCTCATGGTGTGACGGGCGGTGTGTACAAGGCCCGGGAACGTATTCACCGCGGCATGCTGATCCGCGATTACTAGCGATTCCGACTTCGTGTAGGCGAGTTGCAGCCTACAGTCCGAACTGAGATTGGCTTTAAGAGATTAGCTTACCCTCGCGAGTTCGCAACTCGTTGTACCAACCATTGTAGCACGTGTGTAGCCCAGGTCATAAGGGGCATGATGATTTGACGTCGTCCCCACCTTCCTCCGGTTTGTCACCGGCAGTCTTACTAGAGTGCCCAACTTAATGCTGGCAACTAGTCATAAGGGTTGCGCTCGTTGCGGGACTTAACCCAACATCTCACGACACGAGCTGACGACAACCATGCACCACCTGTCATTCCGTCCCCGAAGGGAACTTTCTATCTCTAGAAATAGCAGAAGATGTCAAGACCTGGTAAGGTTCTTCGCGTTGCTTCGAATTAAACCACATGCTCCACCGCTTGTGCGGGCCCCCGTCAATTCCTTTGAGTTTCAACCTTGCGGTCGTACTCCCCAGGCGGAATACTTAATGCGTTAGCTGCGGCACTGAAGGGCGGAAACCCTCCAACACCTAGTATTCATCGTTTACGGCATGGACTACCAGGGTATCTTAATCCTGTTCGGCTACCCCATGCTTTCGGAGCCTTCAGCGTCAATAACTGACCCAGACAGCCGCCTTTCGCCCACTGGTGTTCTTCCAATATATCTACGCAATTTCACCGCTACACCATGGAGTTCCACTGTCCTTCTTCAGCATTCAAAGTTTCCCCAGTTTCCGATGCACTTCCTCGGGTTAAGCCGAGGGCTTTCACATCAGACTTAAAAAACCGCCTGCGCTCGCTTTACGCCCAATAAATCCGGATAACGCTTGCCACCTACGTATTACCGCGGCTGCTGGCACGTAGTTAGCCGTGGCTTTCTGGTTGAATACCGTCAACGTCTGAACAGTTACTCTCAAACGTGTTCTTCTTCAACAACAGAGTTTTACGACCCGAAAGCCTTCTTCACTCACGCGGCGTTGCTCCATCAGACTTGCGTCCATTGTGGAAGATTCCCTACTGCTGCCTCCCGTAGGAGTTTGGGCCGTGTCTCAGTCCCAATGTGGCCGATCAACCTCTCAGTTCGGCTACGTATCATTGCCTTGGTGAGCCATTACCTCACCAACTAGCTAATACGCCGCGGGTCCATCTCTAAGTGACAGCCGAAGCCGCCTTTCAGCCTAAAACCATGTGGTTTTAGGATTTATACGGTATTAGCATCTGTTTCCAAATGTTATCCCCTGCTTAGAGGCAGGTTACCCACGTGTTACTCACCCATCCGCCGCTCACCGCTCTAAAATCACTTGGTGCAAGCACCGCATTCATTTAGTTAAGTTCGCTCGACTGCATGTATAG

>Lactobacillus casei  APW1161E
TCACTTAGACGGCTCGCTCCCTAAAGGGTTACGCCACCGGCTTCGGGTGTTACAAACTCTCATGGTGTGACGGGCGGTGTGTACAAGGCCCGGGAACGTATTCACCGCGGCGTGCTGATCCGCGATTACTAGCGATTCCGACTTCGTGTAGGCGAGTTGCAGCCTACAGTCCGAACTGAGAATGGCTTTAAGAGATTAGCTTGACCTCGCGGTCTCGCAACTCGTTGTACCATCCATTGTAGCACGTGTGTAGCCCAGGTCATAAGGGGCATGATGATTTGACGTCATCCCCACCTTCCTCCGGTTTGTCACCGGCAGTCTTACTAGAGTGCCCAACTAAATGCTGGCAACTAGTCATAAGGGTTGCGCTCGTTGCGGGACTTAACCCAACATCTCACGACACGAGCTGACGACAACCATGCACCACCTGTCATTTTGCCCCCGAAGGGGAAACCTGATCTCTCAGGTGATCAAAAGATGTCAAGACCTGGTAAGGTTCTTCGCGTTGCTTCGAATTAAACCACATGCTCCACCGCTTGTGCGGGCCCCCGTCAATTCCTTTGAGTTTCAACCTTGCGGTCGTACTCCCCAGGCGGAATGCTTAATGGCGTTAGATGCGGCACTGAAAGGCGGAAACCCTCCACACCTAGCATTCATCGTTTACGGCATGGACTACCAGGGTATCTAATCCTGTTCGCTACCCATGCTTTCGAGCCTCAGCGTCAGTTACAGACCAAGACAGCCGCCTTCGCCACTGGTGTTCTTCCAAATATCTACGCATTTCACCGCTCACCATGGAGTTCACTGTCCTCTTCTGCACTCAAGTTCCCAGTTTCCGATGCGCTTCCTCGGTTAAGCCGAGGGCTTTCACATCAGACTTAAAAAACCGCCTGCGCTCGCTTTACGCCCAATAAATCCGGATAACGCTTGCCACCTACGTATTACCGCGGCTGCTGGCACGTAGTTAGCCGTGGCTTTCTGGTTGGATACCGTCACGCCGACAACAGTTACTCTGCCGACCATTCTTCTCCAACAACAGAGTTTTACGACCCGAAAGCCTTCTTCACTCACGCGGCGTTGCTCCATCAGACTTGCGTCCATTGTGGAAGATTCCCTACTGCTGCCTCCCGTAGGAGTTTGGGCCGTGTCTCAGTCCCAATGTGGCCGATCAACCTCTCAGTTCGGCTACGTATCATCGCCTTGGTGAGCCATTACCTCACCAACTAGCTAATACGCCGCGGGTCCATCCAAAAGCGATAGCTTACGCCATCTTTCAGCCAAGAACCATGCGGTTCTTGGATCTATGCGGTATTAGCATCTGTTTCCAAATGTTATCCCCCACTTAAGGGCAGGTTACCCACGTGTTACTCACCCGTCCGCCACTCGTTCCATGTTGAATCTCGGTGCAAGCACCGATCATCAACAGAGAACTCGTTCGACTGCATGTATAGACTGC

>Lactobacillus plantarum APW1553A
GTCACTTAGGCGGCTGGTTCCTAAAGGTTACCCCACCGACTTTGGGTGTTACAAACTCTCATGGTGTGACGGGCGGTGTGTACAAGGCCCGGGAACGTATTCACCGCGGCATGCTGATCCGCGATTACTAGCGATTCCGACTTCATGTAGGCGAGTTGCAGCCTACAATCCGAACTGAGAATGGCTTTAAGAGATTAGCTTACTCTCGCGAGTTCGCAACTCGTTGTACCATCCATTGTAGCACGTGTGTAGCCCAGGTCATAAGGGGCATGATGATTTGACGTCATCCCCACCTTCCTCCGGTTTGTCACCGGCAGTCTCACCAGAGTGCCCAACTTAATGCTGGCAACTGATAATAAGGGTTGCGCTCGTTGCGGGACTTAACCCAACATCTCACGACACGAGCTGACGACAACCATGCACCACCTGTATCCATGTCCCCGAAGGGAACGTCTAATCTCTTAGATTTGCATAGTATGTCAAGACCTGGTAAGGTTCTTCGCGTAGCTTCGAATTAAACCACATGCTCCACCGCTTGTGCGGGCCCCCGTCAATTCCTTTGAGTTTCAGCCTTGCGGCCGTACTCCCCAGGCGGAATGCTTAATGCGTTAGCTGCAGCACTGAAGGGCGGAAACCCTCCAACACTTAGCATTCATCGTTTACGGTATGGACTACCAGGGTATCTAATCCTGTTTGCTACCCATACTTTCGAGCCTCAGCGTCAGTTACAGACCAGACAGCCGCCTTCGCCACTGGTGTTCTTCCATATATCTACGCATTTCACCGCTACACATGGAGTTCCACTGTCCTCTTCTGCACTCAAGTTTCCCAGTTTCCGATGCACTTCTTCCGGTTGAGCCGAAAGCTTTCACATCAGACTTAAAAAACCGCCTGCGCTCGCTTTACGCCCAATAAATCCGGACAACGCTTGCCACTACGTATTACCGCGGCTGCTGGCACGTAGTTAGCCGTGGCTTTCTGGTTAAATACCGTCAATACCTGAACAGTTACTCTCAGATATGTTCTTCTTTAACAACAGAGTTTTACGAGCCGAAACCCTTCTTCACTCACGCGGCGTTGCTCCATCAGACTTTCGTCCATTGTGGAAGATTCCCTACTGCTGCCTCCCGTAGGAGTTTGGGCCGTGTCTCAGTCCCAATGTGGCCGATTACCCTCTCAGGTCGGCTACGTATCATTGCCATGGTGAGCCGTTACCCCACCATCTAGCTAATACGCCGCGGGACCATCCAAAAGTGATAGCCGAAGCCATCTTTCAAACTCGGACCATGCGGTCCAAGTTGTTATGCGGTATTAGCATCTGTTTCCAGGTGTTATCCCCCACTTCTGGGCAGGTTTCCCACGTGTTACTCACCAGTTCGCCACTCACTCAAATGTAAATCATGATGCAAGCACCAATCAATACCAGAGTTCGTTCGACTGCATGTATAGGAG

>2288C
TCACCTTAGGCGGCTGGCTCCAAAAGGTTACCTCACCGACTTCGGGTGTTACAAACTCTCGTGGTGTGACGGGCGGTGTGTACAAGGCCCGGGAACGTATTCACCGCGGCGTGCTGATCCGCGATTACTAGCGATTCCGGCTTCATGTAGGCGAGTTGCAGCCTACAATCCGAACTGAGAGAAGCTTTAAGAGATTAGCTTAGCCTCGCGACTTCGCGACTCGTTGTACTTCCCATTGTAGCACGTGTGTAGCCCAGGTCATAAGGGGCATGATGATTTGACGTCATCCCCACCTTCCTCCGGTTTGTCACCGGCAGTCTTGCTAGAGTGCCCAACTAAATGATGGCAACTAACAATAAGGGTTGCGCTCGTTGCGGGACTTAACCCAACATCTCACGACACGAGCTGACGACAACCATGCACCACCTGTCACTTTGCCCCCGAAGGGGAAGCTCTATCTCTAGAGTGGTCAAAGGATGTCAAGACCTGGTAAGGTTCTTCGCGTTGCTTCGAATTAAACCACATGCTCCACCGCTTGTGCGGGCCCCCGTCAATTCCTTTGAGTTTCAACCTTGCGGTCGTACTCCCCAGGCGGAGTGCTTAATGCGTTAGCTGCAGCACTGAAGGGCGGAAACCCTCCAACACTTAGCACTCATCGTTTACGGCGTGGACTACCAGGGTATCTAATCCTGTTTGCTCCGCACGCTTTCGAGCCTCAGCGTCAGTTACAGACCAGAGAGCCGCCTTCGCCACTGGTGTTCCTCCATATATCTACGCATTTCACCGCTACACATGGAATTCCACTCTCCTCTTCTGCACTCAAGTCTCCCAGTTTCCAATGACCCTCCCCGGTTGAGCCGGGGGCTTTCACATCAGACTTAAGAAACCGCCTGCGCTCGCTTTACGCCCAATAAATCCGGACAACGCTTGCCACCTACGTATTACCGCGGCTGCTGGCACGTAGTTAGCCGTGGCTTTCTGGTTAGATACCGTCAAGGGATGAACAGTTACTCTCATCCTTGTTCTTCTCTAACAACAGAGTTTTACGATCCGAAAACCTTCTTCACTCACGCGGCGTTGCTCGGTCAGACTTTCGTCCATTGCCGAAGATTCCCTACTGCTGCCTCCCGTAGGAGTTTGGGCCGTGTCTCAGTCCCAATGTGGCCGATCACCCTCTCAGGTCGGCTATGCATCGTGGCCTTGGTGAGCCGTTACCTCACCAACTAGCTAATGCACCGCGGGTCCATCCATCAGCGACACCCGAAAGCGCCTTTCAAATCAAAACCATGCGGTTTCGATTGTTATACGGTATTAGCACCTGTTTCCAAGTGTTATCCCCTTCTGATGGGCAGGTTACCCACGTGTTACTCACCCGTTCGCCACTCTTCTTTTTCCGGTGGAGCAAGCTCCGGTGGAAAAAGAAGCGTACGACTGCACGTATAGCGC

>Lactobacillus fermentum APW2288
AATTCTGTCACCTTAGGCGGCTGGCTCCTAAAGGTTACCCCACCGACTTTGGGTGTTACAAACTCTCATGGTGTGACGGGCGGTGTGTACAAGGCCCGGGAACGTATTCACCGCGGCATGCTGATCCGCGATTACTAGCGATTCCAACTTCGTGCAGGCGAGTTGCAGCCTGCAGTCCGAACTGAGAACGGTTTTAAGAGATTTGCTTGCCCTCGCGAGTTCGCGACTCGTTGTACCGTCCATTGTAGCACGTGTGTAGCCCAGGTCATAAGGGGCATGATGATCTGACGTCGTCCCCACCTTCCTCCGGTTTGTCACCGGCAGTCTCACTAGAGTGCCCAACTTAATGCTGGCAACTAGTAACAAGGGTTGCGCTCGTTGCGGGACTTAACCCAACATCTCACGACACGAGCTGACGACGACCATGCACCACCTGTCATTGCGTTCCCGAAGGAAACGCCCTATCTCTAGGGTTGGCGCAAGATGTCAAGACCTGGTAAGGTTCTTCGCGTAGCTTCGAATTAAACCACATGCTCCACCGCTTGTGCGGGCCCCGTCAATTCCTTTGAGTTTCAACCTTGCGGTCGTACTCCCCAGGCGGAGTGCTTAATGCGTTAGCTCCGGCACTGAAGGGCGGAAACCCTCCAACACCTAGCACTCATCGTTTACGGCATGGACTACCAGGGTATCTAATCCTGTTCGCTACCCATGCTTTCGAGTCTCAGCGTCAGTTGCAGACCAGGTAGCCGCCTTCGCCACTGGTGTTCTTCCATATATCTACGCATTCCCACCGCTACACATGGAGTTCCACTACCCTCTTCTGCACTCAAGTTATCCAGTTTCCGATGCACTTCTCCGGGTTAAGCCGAAGGCTTTCACATCAGACTTAGAAAACCGCCTGCACTCTCTTTACGCCCAATAAATCCGGATAACGCTTGCCACCTACGTATTACCGCGGCTGCTGGCACGTAGTTAGCCGTGACTTTCTGGTTAAATACCGTCAACGTATGAACAGTTACTCTCATACGTGTTCTTCTTTAACAACAGAGCTTTACGAGCCGAAACCCTTCTTCACTCACGCGGTGTTGCTCCATCAGGCTTGCGCCCATTGTGGAAGATTCCCTACTGCTGCCTCCCGTAGGAGTATGGGCCGTGTCTCAGTCCCATTGTGGCCGATCAGTCTCTCAACTCGGCTATGCATCATCGCCTTGGTAGGCCGTTACCCCACCAACAAGCTAATGCACCGCAGGTCCATCCAGAAGTGATAGCGAGAAGCCATCTTTTAAGCGTTGTTCATGCGAACAACGTTGTTATGCGGTATTAGCATCTGTTTCCAAATGTTGTCCCCCGCTTCTGGGCAGGTTACCTACGTGTTACTCACCCGTCCGCCACTCGTTGGCGACCAAAATCAATCAGGTGCAAGCACCATCAATCAATTTGGGCCAACGCGTTCGACTGCATGTATAGGCAC

> Lactobacillus casei APW2439D
CTTGTCACTTAGACGGCTCGCTCCCTAAAGGGTTACGCCACCGGCTTCGGGTGTTACAAACTCTCATGGTGTGACGGGCGGTGTGTACAAGGCCCGGGAACGTATTCACCGCGGCGTGCTGATCCGCGATTACTAGCGATTCCGACTTCGTGTAGGCGAGTTGCAGCCTACAGTCCGAACTGAGAATGGCTTTAAGAGATTAGCTTGACCTCGCGGTCTCGCAACTCGTTGTACCATCCATTGTAGCACGTGTGTAGCCCAGGTCATAAGGGGCATGATGATTTGACGTCATCCCCACCTTCCTCCGGTTTGTCACCGGCAGTCTTACTAGAGTGCCCAACTAAATGCTGGCAACTAGTCATAAGGGTTGCGCTCGTTGCGGGACTTAACCCAACATCTCACGACACGAGCTGACGACAACCATGCACCACCTGTCATTTTGCCCCCGAAGGGGAAACCTGATCTCTCAGGTGATCAAAAGATGTCAAGACCTGGTAAGGTTCTTCGCGTTGCTTCGAATTAAACCACATGCTCCACCGCTTGTGCGGGCCCCCGTCAATTCCTTTGAGTTTCAACCTTGCGGTCGTACTCCCCAGGCGGAATGCTTAATGCGTTAGCTGCGGCACKGAAGACGGSGGAAACCCCTACMMASACASTAGTYATTACWTGYKTTTASGGCATGGAAYTACCGAGGGGTATTYTAATCCGTTKTTCGAMTACGCSATGSTTTGSGAGTCYTMAGCTGTCAGATYACAGATCSARACAGCCCGCYYTCGCCCACTGGTGCTYCTYCCAKATATCTACGCATTTCACCGCCTMCACATGGAGTTCCACTGTCCTCTTCTGCACTCAAGTTTCCCAGTTTCCGATGCGCTTCCTCGGTTAAGCCGAGGGCTTTCACATCAGACTTAAAAAACCGCCTGCGCTCGCTTTACGCCCAATAAATCCGGATAACGCTTGCCACCTACGTATTACCGCGGCTGCTGGCACGTAGTTAGCCGTGGCTTTCTGGTTGGATACCGTCACGCCGACAACAGTTACTCTGCCGACCATTCTTCTCCAACAACAGAGTTTTACGACCCGAAAGCCTTCTTCACTCACGCGGCGTTGCTCCATCAGACTTGCGTCCATTGTGGAAGATTCCCTACTGCTGCCTCCCGTAGGAGTTTGGGCCGTGTCTCAGTCCCAATGTGGCCGATCAACCTCTCAGTTCGGCTACGTATCATCGCCTTGGTGAGCCATTACCTCACCAACTAGCTAATACGCCGCGGGTCCATCCAAAAGCGATAGCTTACGCCATCTTTCAGCCAAGAACCATGCGGTTCTTGGATCTATGCGGTATTAGCATCTGTTTCCAAATGTTATCCCCCACTTAAGGGCAGGTTACCCACGTGTTACTCACCCGTCCGCCACTCGTTCCATGTTGAATCTCGGTGCAAGCACCGATCATCAACGAGAACTCGTTCGACTGCATGTATAGACCC

>Lactobacillus casei APW2288E
GTCACTTAGACGGCTCGCTCCCTAAAGGGTTACGCCACCGGCTTCGGGTGTTACAAACTCTCATGGTGTGACGGGCGGTGTGTACAAGGCCCGGGAACGTATTCACCGCGGCGTGCTGATCCGCGATTACTAGCGATTCCGACTTCGTGTAGGCGAGTTGCAGCCTACAGTCCGAACTGAGAATGGCTTTAAGAGATTAGCTTGACCTCGCGGTCTCGCAACTCGTTGTACCATCCATTGTAGCACGTGTGTAGCCCAGGTCATAAGGGGCATGATGATTTGACGTCATCCCCACCTTCCTCCGGTTTGTCACCGGCAGTCTTACTAGAGTGCCCAACTAAATGCTGGCAACTAGTCATAAGGGTTGCGCTCGTTGCGGGACTTAACCCAACATCTCACGACACGAGCTGACGACAACCATGCACCACCTGTCATTTTGCCCCCGAAGGGGAAACCTGATCTCTCAGGTGATCAAAAGATGTCAAGACCTGGTAAGGTTCTTCGCGTTGCTTCGAATTAAACCACATGCTCCACCGCTTGTGCGGGCCCCCGTCAATTCCTTTGAGTTTCAACCTTGCGGTCGTACTCCCCAGCGGAATGCTTAATGCGTTAGCTGCGGCACTGAAAGGGCGGAAACCCTCCAACACCTAGCATTCATCGTTTACGGCATGGACTACCAGGGTATCTATCTGTTCGCTACCCATGCTTTCGAGCCTCAGCGTCAGTTACAGACCAGACAGCCGCCTCGCCACTGGTGTTCTTCCATATATCTACGCATTTCACCGCTACACATGGAGTTCCACTGTCCTCTTCTGCACTCAAAGTTTCCCAGTTTCCGATGCGCTTCCTCGGTTAAGCCGAGGGCTTTCACATCAGACTTAAAAAACCGCCTGCGCTCGCTTTACGCCCAATAAATCCGGATAACGCTTGCCACCTACGTATTACCGCGGCTGCTGGCACGTAGTTAGCCGTGGCTTTCTGGTTGGATACCGTCACGCCGACAACAGTTACTCTGCCGACCATTCTTCTCCAACAACAGAGTTTTACGACCCGAAAGCCTTCTTCACTCACGCGGCGTTGCTCCATCAGACTTGCGTCCATTGTGGAAGATTCCCTACTGCTGCCTCCCGTAGGAGTTTGGGCCGTGTCTCAGTCCCAATGTGGCCGATCAACCTCTCAGTTCGGCTACGTATCATCGCCTTGGTGAGCCATTACCTCACCAACTAGCTAATACGCCGCGGGTCCATCCAAAAGCGATAGCTTACGCCATCTTTCAGCCAAGAACCATGCGGTTCTTGGATCTATGCGGTATTAGCATCTGTTTCCAAATGTTATCCCCCACTTAAGGGCAGGTTACCCACGTGTTACTCACCCGTCCGCCACTCGTTCCATGTTGAATCTCGGTGCAAGCACCGATCATCAACGAGAACTCGTCGACTGCATGTATAGC

>Lactobacillus brevis APW1553C
GTCACCTTAGACGGCTGACTCCCGAAGGTTATCTCACCGGCTTTGGGTGTTACAAACTCTCATGGTGTGACGGGCGGTGTGTACAAGGCCCGGGAACGTATTCACCGCGGCATGCTGATCCGCGATTACTAGCGATTCCAACTTCATGTAGGCGAGTTGCAGCCTACAATCCGAACTGAGAACGGCTTTAAGAGATTAGCTTAGCCTCACGACTTCGCAACTCGTTGTACCGTCCATTGTAGCACGTGTGTAGCCCAGGTCATAAGGGGCATGATGATTTGACGTCATCCCCACCTTCCTCCGGTTTGTCACCGGCAGTCTCACCAGAGTGCCCAACTGAATGCTGGCAACTGATAATAAGGGTTGCGCTCGTTGCGGGACTTAACCCAACATCTCACGACACGAGCTGACGACAACCATGCACCACCTGTCATTCTGTCCCCGAAGGGAACGTCTTATCTCTAAGATTGGCAGAAGATGTCAAGACCTGGTAAGGTTCTTCGCGTAGCTTCGAATTAAACCACATGCTCCACCGCTTGTGCGGGCCCCCGTCAATTCCTTTGAGTTTCAACCTTGCGGTCGTACTCCCCAGGCGGAGTGCTTAATGCGTTAGCTGCAGCACTGAAGGGCGGAAACCCTCCAACACTTAGCACTCATCGTTTACGGCATGGACTACCAGGGTATCTAATCCTGTTCGCTACCCATGCTTTCGAGCCTCAGCGTCAGTTACAGACTAGACAGCCGCCTTCGCCACTGGTGTTCTTCCATATATCTACGCATTCCACCGCTACACATGGAGTTCCACTGTCCTCTTCTGCACTCAAGTCTCCCAGTTTCCGATGCACTTCTCCGGTTAAGCCGAAGGCTTTCACATCAGACTTAAAAAACCGCCTGCGCTCGCTTTACGCCCAATAAATCCGGACAACGCTTGCCACCTACGTATTACCGCGGCTGCTGGCACGTAGTTAGCCGTGGCTTTCTGGTTAAATACCGTCAACCCTTGAACAGTTACTCTCAAAGGTGTTCTTCTTTAACAACAGAGTTTTACGAGCCGAAACCCTTCTTCACTCACGCGGCATTGCTCCATCAGACTTTCGTCCATTGTGGAAGATTCCCTACTGCTGCCTCCCGTAGGAGTTTGGGCCGTGTCTCAGTCCCAATGTGGCCGATTACCCTCTCAGGTCGGGTACGTATCATCGTCTTGGTGGGCCTTTACCTCACCAACTAACTAATACGCCGCGGGATCATCCAGAAGTGATAGCCGAAGCCACCTTTCAAACAAAATCCATGCGGATTTTGTTGTTATACGGTATTAGCACCTGTTTCCAAGTGTTATCCCCTGCTTCTGGGCAGATTTCCCACGTGTTACTCACCAGTTCGCCACTCGCTTCATTGTTAAAATCAGTGCAAGCACGTCATTCAACGGAAGCTCGTTCGACTGCATGTATAGGCTG

>Lactobacillus brevis APW1553D
GTCAGCATGAGACGGCTCGTAGTCCCGTAAGGCTATCTCTCCGGCTTTGGGTGTTACAAACTCTCATGGTGTGACGGGCGGTGTGTACAAGGCCCGGGAACGTATTCACCGCGGCATGCTGATCCGCGATTACTAGCGATTCCAACTTCATGTAGGCGAGTTGCAGCCTACAATCCGAACTGAGAACGGCTTTAAGAGATTAGCTTAGCCTCACGACTTCGCGACTCGTTGTACCGTCCATTGTAGCACGTGTGTAGCCCAGGTCATAAGGGGCATGATGATTTGACGTCATCCCCACCTTCCTCCGGTTTGTCACCGGCAGTCTCACCAGAGTGCCCAACTGAATGCTGGCAACTGATAATAAGGGTTGCGCTCGTTGCGGGACTTAACCCAACATCTCACGACACGAGCTGACGACAACCATGCACCACCTGTCATTCTGTCCCCGAAGGGAACGTCTTATCTCTAAGATTGGCAGAAGATGTCAAGACCTGGTAAGGTTCTTCGCGTAGCTTCGAATTAAACCACATGCTCCACCGCTTGTGCGGGCCCCCGTCAATTCCTTTGAGTTTCAACCTTGCGGTCGTACTCCCCAGGCGGAGTGCTTAATGCGTTAGCTGCAGCACTGAAGGGCGGAAACCCTCCAACACTTAGCACTCATCGTTTACGGCATGGACTACCAGGGTATCTAATCCTGTTCGCTACCCATGCTTTCGAGCCTCAGCGTCAGTTACAGACTAGACAGCCGCCTTCGCCACTGGTGTTCTTCCATATATCTACGCATTCCACCGCTACACATGGAGTTCCACTGTCCTCTTCTGCACTCAAGTCTCCCAGTTTCCGATGCACTTCTCCGGTTAAGCCGAAGGCTTTCACATCAGACTTAAAAAACCGCCTGCGCTCGCTTTACGCCCAATAAATCCGGACAACGCTTGCCACCTACGTATTACCGCGGCTGCTGGCACGTAGTTAGCCGTGGCTTTCTGGTTAAATACCGTCAACCCTTGAACAGTTACTCTCAAAGGTGTTCTTCTTTAACAACAGAGTTTTACGAGCCGAAACCCTTCTTCACTCACGCGGCATTGCTCCATCAGACTTTCGTCCATTGTGGAAGATTCCCTACTGCTGCCTCCCGTATAGATTTGGGCCGTGTCTCAGTCCCAATGTGGCCGATTACCCTCTCAGGTCGGCTACGTATCATCGTCTTGGTGGGCCTTTACCTCACCAACTAACTAATACGCCGCGGGATCATCCAGAAGTGATAGCCGAAGCCACCTTTCAAACAAAATCCATGCGGATTTTGTTGTTATACGGTATTAGCACCTGTTTCCAAGTGTTATCCCCTGCTTCTGGGCAGATTTCCCACGTGTTACTCACCAGTTCGCCACTCGCTTCATTGTTGAAATCAGTGCAAGCACGTCATTCACACGGAAGCTCGTTCGACTGCATGTATGAG

>Lactobacillus plantarum APW1634E
GTCACCTTAGGCGGCTGGTTCCTAAAAGGTTACCCCACCGACTTTGGGTGTTACAAACTCTCATGGTGTGACGGGCGGTGTGTACAAGGCCCGGGAACGTATTCACCGCGGCATGCTGATCCGCGATTACTAGCGATTCCGACTTCATGTAGGCGAGTTGCAGCCTACAATCCGAACTGAGAATGGCTTTAAGAGATTAGCTTACTCTCGCGAGTTCGCAACTCGTTGTACCATCCATTGTAGCACGTGTGTAGCCCAGGTCATAAGGGGCATGATGATTTGACGTCATCCCCACCTTCCTCCGGTTTGTCACCGGCAGTCTCACCAGAGTGCCCAACTTAATGCTGGCAACTGATAATAAGGGTTGCGCTCGTTGCGGGACTTAACCCAACATCTCACGACACGAGCTGACGACAACCATGCACCACCTGTATCCATGTCCCCGAAGGGAACGTCTAATCTCTTAGATTTGCATAGTATGTCAAGACCTGGTAAGGTTCTTCGCGTAGCTTCGAATTAAACCACATGCTCCACCGCTTGTGCGGGCCCCCGTCAATTCCTTTGAGTTTCAGCCTTGCGGCCGTACTCCCCAGGCGGAATGCTTAATGCGTTAGCTGCAGCACTGAAGGGCGGAAACCCTCCAACACTTAGCATTCATCGTTTACGGTATGGACTACCAGGGTATCTAATCCTGTTTGCTACCCATACTTTCGAGCCTCAGCGTCAGTTACAGACCAGACAGCCGCCTTCGCCACTGGTGTTCTTCCATATATCTACGCATTTCACCGCTACACATGGAGTTCCACTGTCCTCTTCTGCACTCAAGTTTCCCAGTTTCCGATGCACTTCTTCGGTTGAGCCGAAGGCTTTCACATCAGACTTAAAAAACCGCCTGCGCTCGCTTTACGCCCAATAAATCCGGACAACGCTTGCCACCTACGTATTACCGCGGCTGCTGGCACGTAGTTAGCCGTGGCTTTCTGGTTAAATACCGTCAATACCTGAACAGTTACTCTCAGATATGTTCTTCTTTAACAACAGAGTTTTACGAGCCGAAACCCTTCTTCACTCACGCGGCGTTGCTCCATCAGACTTTCGTCCATTGTGGAAGATTCCCTACTGCTGCCTCCCGTATGAGTTTGGGCCGTGTCTCAGTCCCAATGTGGCCGATTACCCTCTCAGGTCGGCTACGTATCATTGCCATGGTGAGCCGTTACCCCACCATCTAGCTAATACGCCGCGGGACCATCCAAAAGTGATAGCCGAAGCCATCTTTCAAACTCGGACCATGCGGTCCAAGTTGTTATGCGGTATTAGCATCTGTTTCCAGGTGTTATCCCCCGCTTCTGGGCAGGTTTCCCACGTGTTACTCACCAGTTCGCCACTCACTCAAATGTAAATCATGATGCAAGCACCAATCAATACCAGAGTTCGTTCGACTGCATGTATAGCAT

> Lactobacillus brevis APW2112
GTCACTTAGACGGCTGACTCCCGAAGGTTATCTCACCGGCTTTGGGTGTTACAAACTCTCATGGTGTGACGGGCGGTGTGTACAAGGCCCGGGAACGTATTCACCGCGGCATGCTGATCCGCGATTACTAGCGATTCCAACTTCATGTAGGCGAGTTGCAGCCTACAATCCGAACTGAGAACGGCTTTAAGAGATTAGCTTAGCCTCACGACTTCGCAACTCGTTGTACCGTCCATTGTAGCACGTGTGTAGCCCAGGTCATAAGGGGCATGATGATTTGACGTCATCCCCACCTTCCTCCGGTTTGTCACCGGCAGTCTCACCAGAGTGCCCAACTGAATGCTGGCAACTGATAATAAGGGTTGCGCTCGTTGCGGGACTTAACCCAACATCTCACGACACGAGCTGACGACAACCATGCACCACCTGTCATTCTGTCCCCGAAGGGAACGTCTTATCTCTAAGATTGGCAGAAGATGTCAAGACCTGGTAAGGTTCTTCGCGTAGCTTCGAATTAAACCACATGCTCCACCGCTTGTGCGGGCCCCCGTCAATTCCTTTGAGTTTCAACCTTGCGGTCGTACTCCCCAGGCGGAGTGCTTAATGCGTTAGCTGCAGCACTGAAGGGCGGAAACCCTCCAACACTTAGCACTCATCGTTTACGGCATGGACTACCAGGGTATCTAATCCTGTTCGCTACCCATGCTTTCGAGCCTCAGCGTCAGTTACAGACTAGACAGCCGCCTTCGCCACTGGTGTTCTTCCATATATCTACGCATTCCACCGCTACACATGGAGTTCCACTGTCCTCTTCTGCACTCAAGTCTCCCAGTTTCCGATGCACTTCTCCGGTTAAGCCGAAAGCTTTCACATCAGACTTAAAAAACCGCCTGCGCTCGCTTTACGCCCAATAAATCCCGGACAACGCTTGCCACATACGTATTACCGCGGCTGCTGGCACGTAGTTAGCCGTGGCTTTCTGGTTAAATACCGTCAACCCTTGAACAGTTACTCTCAAAGGTGTTCTTCTTTAACAACAGAGTTTTACGAGCCGAAACCCTTCTTCACTCACGCGGCATTGCTCCATCAGACTTTCGTCCATTGTGGAAGATTCCCTACTGCTGCCTCCCGTAGGAGTTTGGGCCGTGTCTCAGTCCCAATGTGGCCGATTACCCTCTCAGGTGGGCTACGTATCATCGTCTTGGTGGGCCTTTACCTCACCAACTAACTAATACGCCGCGGGATCATCCAGAAGTGATAGCCGAAGCCACCTTTCAAACAAAATCCATGCGGATTTTGTTGTTATACGGTATTAGCACCTGTTTCCAAGTGTTATCCCCTGCTTCTGGGCAGATTTCCCACGTGTTACTCACCAGTTCGCCACTCGCTTCATTGTTAAAATCAGTGCAAGCACGTCATTCAACGGAAGCTCGTTCGACTGCATGTATAGGCATGCC

>Lactobacillus casei APW2172A
TTGTCACTTAGACGGCTCGCTCCCTAAAGGGTTACGCCACCGGCTTCGGGTGTTACAAACTCTCATGGTGTGACGGGCGGTGTGTACAAGGCCCGGGAACGTATTCACCGCGGCGTGCTGATCCGCGATTACTAGCGATTCCGACTTCGTGTAGGCGAGTTGCAGCCTACAGTCCGAACTGAGAATGGCTTTAAGAGATTAGCTTGACCTCGCGGTCTCGCAACTCGTTGTACCATCCATTGTAGCACGTGTGTAGCCCAGGTCATAAGGGGCATGATGATTTGACGTCATCCCCACCTTCCTCCGGTTTGTCACCGGCAGTCTTACTAGAGTGCCCAACTAAATGCTGGCAACTAGTCATAAGGGTTGCGCTCGTTGCGGGACTTAACCCAACATCTCACGACACGAGCTGACGACAACCATGCACCACCTGTCATTTTGCCCCCGAAGGGGAAACCTGATCTCTCAGGTGATCAAAAGATGTCAAGACCTGGTAAGGTTCTTCGCGTTGCTTCGAATTAAACCACATGCTCCACCGCTTGTGCGGGCCCCCGTCAATTCCTTTGAGTTTCAACCTTGCGGTCGTACTCCCCAGGCGGAATGCTTAATGCGTTAGCTGCGGCACTGAAGGGCGGAAACCCTCCAACACCTAGCATTCATCGTTTACGGCATGGACTACCAGGGTATCTAATCCTGTTCGCTACCCATGCTTTCGAGCCTCAGCGTCAGTTACAGACCAGACAGCCGCCTTCGCCACTGGTGTTCTTCCATATATCTACGCATTTCACCGCTACACATGGAGTTCCACTGTCCTCTTCTGGACTCAAGTTTCCCAGTTTCCGATGCGCTTCCTCGGTTAAGCCGAGGCTTCACATCAGACTTAAAAACCGCTGCGCTCGCTTTACGCCCAATAAATCCGGATAACGCTTGCCACTACGTATTACCGCGGCTGCTGGCACGTAGTTAGCCGTGGCTTTCTGGTTGGATACCGTCACGCCGACAACAGTTACTCTGCCGACCATTCTTCTCCAACAACAGAGTTTTACGACCCGAAAGCCTTCTTCACTCACGCGGCGTTGCTCCATCAGACTTGCGTCCATTGTGGAAGATTCCCTACTGCTGCCTCCCGTAGGAGTTTGGGCCGTGTCTCAGTCCCAATGTGGCCGATCAACCTCTCAGTTCGGCTACGTATCATCGCCTTGGTGAGCCATTACCTCACCAACTAGCTAATACGCCGCGGGTCCATCCAAAAGCGATAGCTTACGCCATCTTTCAGCCAAGAACCATGCGGTTCTTGGATCTATGCGGTATTAGCATCTGTTTCCAAATGTTATCCCCCACTTAAGGGCAGGTTACCCACGTGTTACTCACCCGTCCGCCACTCGTTCCATGTTGAATCTCGGTGCAAGCACCGATCATCAACGAGAACTCGTTCGACTGCATGTATAGAAGCC

>Lactobacillus (para)buchneri APW2213E
ACTTAGACGGCTGGTCCCCGAAGGTTACCTCACCGGCTTTGGGTGTTACAAACTCTCATGGTGTGACGGGCGGTGTGTACAAGGCCCGGGAACGTATTCACCGTGGCATGCTGATCCACGATTACTAGCGATTCCAACTTCATGTAGGCGAGTTGCAGCCTACAATCCGAACTGAGAACGGCTTTAAGAGATTAGCTTGACCTCGCGGTTTCGCGACTCGTTGTACCGTCCATTGTAGCACGTGTGTAGCCCAGGTCATAAGGGGCATGATGATTTGACGTCATCCCCACCTTCCTCCGGTTTGTCACCGGCAGTCTTGCTAGAGTGCCCAACTGAATGCTGGCAACTAACAATAAGGGTTGCGCTCGTTGCGGGACTTAACCCAACATCTCACGACACGAGCTGACGACAACCATGCACCACCTGTCATTCTGTCCCCGAAGGGAACGCCTAATCTCTTAGGTTGGCAGAAGATGTCAAGACCTGGTAAGGTTCTTCGCGTAGCATCGAATTAAACCACATGCTCCACCGCTTGTGCGGGCCCCCGTCAATTCCTTTGAGTTTCAACCTTGCGGTCGTACTCCCCAGGCGGAGTGCTTAATGCGTTAGCTGCAGCACTGAAGGGCGGAAACCCTCCAACACTTAGCACTCATCGTTTACGGCATGGACTACCAGGGTATCTAATCCTGTTCGCTACCCATGCTTTCGAGCCTCAGCGTCAGTTACAGACCAGACAGCCGCCTTCGCCACTGGTGTTCTTCCATATATCTACGCATTTCACCGCTACACATGGAGTTCCACTGTCCTCTTCTGCACTCAAGTCTCCTGGTTTCCGATGCACTTCTCCGGGTTAAGCCGAAGGCTTTCACATCAGACCTAAGAAACCGCCTGCGCTCGCTTTACGCCCAATAAATCCGGACAACGCTTGCCACCTACGTATTACCGCGGCTGCTGGCACGTAGTTAGCCGTGGCTTTCTGGTTGGATACCGTCAAGATGTGAACAGTTACTCTCACACCTGTTCTTCTCCAACAACAGAGTTTTACGAGCCGAAACCCTTCATCACTCACGCGGCGTTGCTCCATCAGACTTTCGTCCATTGTGGAAGATTCCCTACTGCTGCCTCCCGTAGGAGTTTGGGCCGTGTCTCAGTCCCAATGTGGCCGATTACCCTCTCAGGTCGGCTACGTATCATTGCCTTGGTAGGCCGTTACCTTACCAACAAGCTAATACGCCGCGGGTCCATCCTAAAGTGATAGCCGAAGCCATCTTTTAAACCAAAACCAGGTGGTTTTGGTTGTTATACGGTATTAGCACCTGTTTCCAAGTGTTATCCCCTACTTCAAGGGCAGGTTACCCACGTGTTACTCACCAGTTCGCCACTCGTCTCAATGTTAAATCAGTTAAATGCAAGCATCTCTAACATCAATAACCAAGACGCGTCGACTGCATGTATAGGACC

>Lactobacillus paracasei APW1161D
TAGACGGCTCGCTCCCTAAAGGGTTACGCCACCGGCTTCGGGTGTTACAAACTCTCATGGTGTGACGGGCGGTGTGTACAAGGCCCGGGAACGTATTCACCGCGGCGTGCTGATCCGCGATTACTAGCGATTCCGACTTCGTGTAGGCGAGTTGCAGCCTACAGTCCGAACTGAGAATGGCTTTAAGAGATTAGCTTGACCTCGCGGTCTCGCAACTCGTTGTACCATCCATTGTAGCACGTGTGTAGCCCAGGTCATAAGGGGCATGATGATTTGACGTCATCCCCACCTTCCTCCGGTTTGTCACCGGCAGTCTTACTAGAGTGCCCAACTAAATGCTGGCAACTAGTCATAAGGGTTGCGCTCGTTGCGGGACTTAACCCAACATCTCACGACACGAGCTGACGACAACCATGCACCACCTGTCATTTTGCCCCCGAAGGGGAAACCTGATCTCTCAGGTGATCAAAAGATGTCAAGACCTGGTAAGGTTCTTCGCGTTGCTTCGAATTAAACCACATGCTCCACCGCTTGTGCGGGCCCCCGTCAATTCCTTTGAGTTTCAACCTTGCGGTCGTACTCCCCAGGCGGAATGCTTAATGCGTTAGCTGCGGCACTGAAGGGCGGAAACCCTCCAACACCTAGCATTCATCGTTTACGGCATGGACTACCAGGGTATCTAATCCTGTTCGCTACCCATGCTTTCGAGCCTCAGCGTCAGTTACAGACCAGACAGCCGCCTTCGCCACTGGTGTTCTTCCATATATCTACGCATTTCACCGCTACACATGGAGTTCCACTGTCCTCTTCTGCACTCAAGTTTCCCAGTTTCCGATGCGCTTCCTCGGTTAAGCCGAGGGCTTTCACATCAGACTTAAAAAACCGCCTGCGCTCGCTTTACGCCCAATAAATCCGGATAACGCTTGCCACCTACGTATTACCGCGGCTGCTGGCACGTAGTTAGCCGTGGCTTTCTGGTTGGATACCGTCACGCCGACAACAGTTACTCTGCCGACCATTCTTCTCCAACAACAGAGTTTTACGACCCGAAAGCCTTCTTCACTCACGCGGCGTTGCTCCATCAGACTTGCGTCCATTGTGGAAGATTCCCTACTGCTGCCTCCCGTAGGAGTTTGGGCCGTGTCTCAGTCCCAATGTGGCCGATCAACCTCTCAGTTCGGCTACGTATCATCGCCTTGGTGAGCCATTACCTCACCAACTAGCTAATACGCCGCGGGTCCATCCAAAAGCGATAGCTTACGCCATCTTTCAGCCAAGAACCATGCGGTTCTTGGATCTATGCGGTATTAGCATCTGTTTCCAAATGTTATCCCCCACTTAAGGGCAGGTTACCCACGTGTTACTCACCCGTCCGCCACTCGTTCCATGTTGAATCTCGGTGCAAGCACCGATCATCAACGAGAACTCGTCGACTGCATGTATAGCAG

>Lactobacillus fermentum APW1317D
ACGCGGCTGGCTCCTAAAGGTTACCCCACCGACTTTGGGTGTTACAAACTCTCATGGTGTGACGGGCGGTGTGTACAAGGCCCGGGAACGTATTCACCGCGGCATGCTGATCCGCGATTACTAGCGATTCCGACTTCGTGCAGGCGAGTTGCAGCCTGCAGTCCGAACTGAGAACGGTTTTAAGAGATTTGCTTGCCCTCGCGAGTTCGCGACTCGTTGTACCGTCCATTGTAGCACGTGTGTAGCCCAGGTCATAAGGGGCATGATGATCTGACGTCGTCCCCACCTTCCTCCGGTTTGTCACCGGCAGTCTCACTAGAGTGCCCAACTTAATGCTGGCAACTAGTAACAAGGGTTGCGCTCGTTGCGGGACTTAACCCAACATCTCACGACACGAGCTGACGACAACCATGCACCACCTGTCATTGCGTTCCCGAAGGAAACGCCCTATCTCTAGGGTTGGCGCAAGATGTCAAGACCTGGTAAGGTTCTTCGCGTAGCTTCGAATTAAACCACATGCTCCACCGCTTGTGCGGGCCCCCGTCAATTCCTTTGAGTTTCAACCTTGCGGTCGTACTCCCCAGGCGGAGTGCTTAATGCGTTAGCTCCGGCACTGAAGGGCGGAAACCCTCCAACACCTAGCACTCATCGTTTACGGCATGGACTACCAGGGTATCTAATCCTGTTCGCTACCCATGCTTTCGAGTCTCAGCGTCAGTTGCAGACCAGGTAGCCGCCTTCGCCACTGGTGTTCTTCCATATATCTACGCATTCCACCGCTACACATGGAGTTCCACTACCCTCTTCTGCACTCAAGTTATCCAGTTTCCGATGCACTTCTCCGGTTAAGCCGAAGGCTTTCACATCAGACTTAGAAAACCGCCTGCACTCTCTTTACGCCCAATAAATCCGGATAACGCTTGCCACCTACGTATTACCGCGGCTGCTGGCACGTAGTTAGCCGTGACTTTCTGGTTAAATACCGTCAACGTATGAACAGTTAATCTCATACGTGTTCTTCTTTAACAACAGAGCTTTACGAGCCGAAACCCTTCTTCACTCACGCGGTGTTGCTCCATCAGGCTTGCGCCCATTGTGGAAGATTCCCTACTGCTGCCTCCCGTAGGAGTATGGGCCGTGTCTCAGTCCCATTGTGGCCGATCAGTCTCTCAACTCGGCTATGCATCATCGCCTTGGTAGGCCGTTACCCCACCAACAAGCTAATGCACCGCAGGTCCATCCAGAAGTGATAGCGAGAAGCCATCTTTTAAGCGTTGTTCATGCGAACAACGTTGTTATGCGGTATTAGCATCTGTTTCCAAATGTTGTCCCCCGCTTCTGGGCAGGTTACCTACGTGTTACTCACCCGTCCGCCACTCGTTGGCGACCAAAATCAATCAGGTGCAAGCACCATCAATCAATTCTGGGCCAACGCGTCGACTGCATGTATAG

>Lactobacillus plantarum APW1434B
CCTTAGGCGGCTGATTCCTAAAGGTTACCCCACCGACTTTGGGTGTTACAAACTCTCATGGTGTGACGGGCGGTGTGTACAAGGCCCGGGAACGTATTCACCGCGGCATGCTGATCCGCGATTACTAGCGATTCCGACTTCATGTAGGCGAGTTGCAGCCTACAATCCGAACTGAGAATGGCTTTAAGAGATTAGCTTACTCTCGCGAGTTCGCAACTCGTTGTACCATCCATTGTAGCACGTGTGTAGCCCAGGTCATAAGGGGCATGATGATTTGACGTCATCCCCACCTTCCTCCGGTTTGTCACCGGCAGTCTCACCAGAGTGCCCAACTTAATGCTGGCAACTGATAATAAGGGTTGCGCTCGTTGCGGGACTTAACCCAACATCTCACGACACGAGCTGACGACAACCATGCACCACCTGTATCCATGTCCCCGAAGGGAACGTCTAATCTCTTAGATTTGCATAGTATGTCAAGACCTGGTAAGGTTCTTCGCGTAGCTTCGAATTAAACCACATGCTCCACCGCTTGTGCGGGCCCCCGTCAATTCCTTTGAGTTTCAGCCTTGCGGCCGTACTCCCCAGGCGGAATGCTTAATGCGTTAGCTGCAGCACTGAAGGGCGGAAACCCTCCAACACTTAGCATTCATCGTTTACGGTATGGACTACCAGGGTATCTAATCCTGTTTGCTACCCATACTTTCGAGCCTCAGCGTCAGTTACAGACCAGACAGCCGCCTTCGCCACTGGTGTTCTTCCATATATCTACGCATTTCACCGCTACACATGGAGTTCCACTGTCCTCTTCTGCACTCAAGTTTCCCAGTTTCCGATGCACTTCTTCGGTTGAGCCGAAGGCTTTCACATCAGACTTAAAAAACCGCCTGCGCTCGCTTTACGCCCAATAAATCCGGACAACGCTTGCCACCTACGTATTACCGCGGCTGCTGGCACGTAGTTAGCCGTGGCTTTCTGGTTAAATACCGTCAATACCTGAACAGTTACTCTCAGATATGTTCTTCTTTAACAACAGAGTTTTACGAGCCGAAACCCTTCTTCACTCACGCGGCGTTGCTCCATCAGACTTTCGTCCATTGTGGAAGATTCCCTACTGCTGCCTCCCGTAGGAGTTTGGGCCGTGTCTCAGTCCCAATGTGGCCGATTACCCTCTCAGGTCGGCTACGTATCATTGCCATGGTGAGCCGTTACCTCACCATCTAGCTAATACGCCGCGGGACCATCCAAAAGTGATAGCCGAAGCCATCTTTCAAACTCGGACCATGCGGTCCAAGTTGTTATGCGGTATTAGCATCTGTTTCCAGGTGTTATCCCCCGCTTCTGGGCAGGTTTCCCACGTGTTACTCACCAGTTCGCCACTCACTCAAATGTAAATCATGATGCAAGCACCAATCAATACCAGAGTTCGTCGACTGCATGTATAGCA

>Lactobacillus brevis APW1553B
AGACTGCGCTCGTAGTCCCGTAGGTTATCTCTCCGGCTTTGGGTGTTACAAACTCTCATGGTGTGACGGGCGGTGTGTACAAGGCCCGGGAACGTATTCACCGCGGCATGCTGATCCGCGATTACTAGCGATTCCAACTTCATGTAGGCGAGTTGCAGCCTACAATCCGAACTGAGAACGGCTTTAAGAGATTAGCTTAGCCTCACGACTTCGCGACTCGTTGTACCGTCCATTGTAGCACGTGTGTAGCCCAGGTCATAAGGGGCATGATGATTTGACGTCATCCCCACCTTCCTCCGGTTTGTCACCGGCAGTCTCACCAGAGTGCCCAACTGAATGCTGGCAACTGATAATAAGGGTTGCGCTCGTTGCGGGACTTAACCCAACATCTCACGACACGAGCTGACGACAACCATGCACCACCTGTCATTCTGTCCCCGAAGGGAACGTCTTATCTCTAAGATTGGCAGAAGATGTCAAGACCTGGTAAGGTTCTTCGCGTAGCTTCGAATTAAACCACATGCTCCACCGCTTGTGCGGGCCCCCGTCAATTCCTTTGAGTTTCAACCTTGCGGTCGTACTCCCCAGGCGGAGTGCTTAATGCGTTAGCTGCAGCACTGAAGGGCGGAAACCCTCCAACACTTAGCACTCATCGTTTACGGCATGGACTACCAGGGTATCTAATCCTGTTCGCTACCCATGCTTTCGAGCCTCAGCGTCAGTTACAGACTAGACAGCCGCCTTCGCCACTGGTGTTCTTCCATATATCTACGCATTCCACCGCTACACATGGAGTTCCACTGTCCTCTTCTGCACTCAAGTCTCCCAGTTTCCGATGCACTTCTCCGGTTAAGCCGAAGGCTTTCACATCAGACTTAAAAAACCGCCTGCGCTCGCTTTACGCCCAATAAATCCGGACAACGCTTGCCACCTACGTATTACCGCGGCTGCTGGCACGTAGTTAGCCGTGGCTTTCTGGTTAAATACCGTCAACCCTTGAACAGTTACTCTCAAAGGTGTTCTTCTTTAACAACAGAGTTTTACGAGCCGAAACCCTTCTTCACTCACGCGGCATTGCTCCATCAGACTTTCGTCCATTGTGGAAGATTCCCTACTGCTGCCTCCCGTAGGAGTTTGGGCCGTGTCTCAGTCCCAATGTGGCCGATTACCCTCTCAGGTCGGCTACGTATCATCGTCTTGGTGGGCCTTTACCTCACCAACTAACTAATACGCCGCGGGATCATCCAGAAGTGATAGCCGAAGCCACCTTTCAAACAAAATCCATGCGGATTTTGTTGTTATACGGTATTAGCACCTGTTTCCAAGTGTTATCCCCTGCTTCTGGGCAGATTTCCCACGTGTTACTCACCAGTTCGCCACTCGCTTCATTGTTGAAATCAGTGCAAGCACGTCATTCACAGCGGAACGCTCGTACGACTGTCATGTATATGGC

>Lactobacillus brevis APW1553E
CACTTAGACGGCTGACTCCCGAAGGTTATCTCTCCGGCTTTGGGTGTTACAAACTCTCATGGTGTGACGGGCGGTGTGTACAAGGCCCGGGAACGTATTCACCGCGGCATGCTGATCCGCGATTACTAGCGATTCCAACTTCATGTAGGCGAGTTGCAGCCTACAATCCGAACTGAGAACGGCTTTAAGAGATTAGCTTAGCCTCACGACTTCGCAACTCGTTGTACCGTCCATTGTAGCACGTGTGTAGCCCAGGTCATAAGGGGCATGATGATTTGACGTCATCCCCACCTTCCTCCGGTTTGTCACCGGCAGTCTCACCAGAGTGCCCAACTGAATGCTGGCAACTGATAATAAGGGTTGCGCTCGTTGCGGGACTTAACCCAACATCTCACGACACGAGCTGACGACAACCATGCACCACCTGTCATTCTGTCCCCGAAGGGAACGTCTTATCTCTAAGATTGGCAGAAGATGTCAAGACCTGGTAAGGTTCTTCGCGTAGCTTCGAATTAAACCACATGCTCCACCGCTTGTGCGGGCCCCCGTCAATTCCTTTGAGTTTCAACCTTGCGGTCGTACTCCCCAGGCGGAGTGCTTAATGCGTTAGCTGCAGCACTGAAGGGCGGAAACCCTCCAACACTTAGCACTCATCGTTTACGGCATGGACTACCAGGGTATCTAATCCTGTTCGCTACCCATGCTTTCGAGCCTCAGCGTCAGTTACAGACTAGACAGCCGCCTTCGCCACTGGTGTTCTTCCATATATCTACGCATTCCACCGCTACACATGGAGTTCCACTGTCCTCTTCTGCACTCAAGTCTCCCAGTTTCCGATGCACTTCTCCGGTTAAGCCGAAGGCTTTCACATCAGACTTAAAAAACCGCCTGCGCTCGCTTTACGCCCAATAAATCCGGACAACGCTTGCCACCTACGTATTACCGCGGCTGCTGGCACGTAGTTAGCCGTGGCTTTCTGGTTAAATACCGTCAACCCTTGAACAGTTACTCTCAAAGGTGTTCTTCTTTAACAACAGAGTTTTACGAGCCGAAACCCTTCTTCACTCACGCGGCATTGCTCCATCAGACTTTCGTCCATTGTGGAAGATTCCCTACTGCTGCCTCCCGTAGGAGTTTGGGCCGTGTCTCAGTCCCAATGTGGCCGATTACCCTCTCAGGTGGGCTACGTATCATCGTCTTGGTGGGCCTTTACCTCACCAACTAACTAATACGCCGCGGGATCATCCAGAAGTGATAGCCGAAGCCACCTTTCAAACAAAATCCATGCGGATTTTGTTGTTATACGGTATTAGCACCTGTTTCCAAGTGTTATCCCCTGCTTCTGGGCAGATTTCCCACGTGTTACTCACCAGTTCGCCACTCGCTTCATTGTTAAAATCAGTGCAAGCACGTCATTCACACGGAAGCTCGTTCGACTGCATGTATAGGC

>Lactobacillus plantarum APW1634A
CATTAAGCGGCTATGTTCCTAAAGAGTTACCCCACCGACTTTGGGTGTTACAAACTCTCATGGTGTGACGGGCGGTGTGTACAAGGCCCGGGAACGTATTCACCGCGGCATGCTGATCCGCGATTACTAGCGATTCCGACTTCATGTAGGCGAGTTGCAGCCTACAATCCGAACTGAGAATGGCTTTAAGAGATTAGCTTACTCTCGCGAGTTCGCAACTCGTTGTACCATCCATTGTAGCACGTGTGTAGCCCAGGTCATAAGGGGCATGATGATTTGACGTCATCCCCACCTTCCTCCGGTTTGTCACCGGCAGTCTCACCAGAGTGCCCAACTGAATGCTGGCAACTGATAATAAGGGTTGCGCTCGTTGCGGGACTTAACCCAACATCTCACGACACGAGCTGACGACAACCATGCACCACCTGTATCCATGTCCCCGAAGGGAACGTCTAATCTCTTAGATTTGCATAGTATGTCAAGACCTGGTAAGGTTCTTCGCGTAGCTTCGAATTAAACCACATGCTCCACCGCTTGTGCGGGCCCCCGTCAATTCCTTTGAGTTTCAGCCTTGCGGCCGTACTCCCCAGGCGGAATGCTTAATGCGTTAGCTGCAGCACTGAAGGGCGGAAACCCTCCAACACTTAGCATTCATCGTTTACGGTATGGACTACCAGGGTATCTAATCCTGTTTGCTACCCATACTTTCGAGCCTCAGCGTCAGTTACAGACCAGACAGCCGCCTTCGCCACTGGTGTTCTTCCATATATCTACGCATTTCACCGCTACACATGGAGTTCCACTGTCCTCTTCTGCACTCAAGTTTCCCAGTTTCCGATGCACTTCTTCGGTTGAGCCGAAGGCTTTCACATCAGACTTAAAAAACCGCCTGCGCTCGCTTTACGCCCAATAAATCCGGACAACGCTTGCCACCTACGTATTACCGCGGCTGCTGGCACGTAGTTAGCCGTGGCTTTCTGGTTAAATACCGTCAATACCTGAACAGTTACTCTCAGATATGTTCTTCTTTAACAACAGAGTTTTACGAGCCGAAACCCTTCTTCACTCACGCGGCGTTGCTCCATCAGACTTTCGTCCATTGTGGAAGATTCCCTACTGCTGCCTCCCGTAGGAGTTTGGGCCGTGTCTCAGTCCCAATGTGGCCGATTACCCTCTCAGGTCGGCTACCTATCATTGCCATGGTGAGCCGTTACCTCACCATCTAGCTAATACGCCGCGGGACCATCCAAAAGTGATAGCCGAAGCCATCTTTCAAGCTCGGACCATGCGGTCCAAGTTGTTATGCGGTATTAGCATCTGTTTCCAGGTGTTATCCCCCGCTTCTGGGCAGGTTTCCCACGTGTTACTCACCAGTTCGCCACTCACTCAAATGTAAATCATGATGCAAGCACCAATCATATACCAGAGTTCGTTCGTACTGCTATCTATATACAG

>Lactobacillus plantarum APW1634B
TAAAGGTTACCCCACCGACTTTGGGTGTTACAAACTCTCATGGTGTGACGGGCGGTGTGTACAAGGCCCGGGAACGTATTCACCGCGGCATGCTGATCCGCGATTACTAGCGATTCCGACTTCATGTAGGCGAGTTGCAGCCTACAATCCGAACTGAGAATGGCTTTAAGAGATTAGCTTACTCTCGCGAGTTCGCAACTCGTTGTACCATCCATTGTAGCACGTGTGTAGCCCAGGTCATAAGGGGCATGATGATTTGACGTCATCCCCACCTTCCTCCGGTTTGTCACCGGCAGTCTCACCAGAGTGCCCAACTTAATGCTGGCAACTGATAATAAGGGTTGCGCTCGTTGCGGGACTTAACCCAACATCTCACGACACGAGCTGACGACAACCATGCACCACCTGTATCCATGTCCCCGAAGGGAACGTCTAATCTCTTAGATTTGCATAGTATGTCAAGACCTGGTAAGGTTCTTCGCGTAGCTTCGAATTAAACCACATGCTCCACCGCTTGTGCGGGCCCCCGTCAATTCCTTTGAGTTTCAGCCTTGCGGCCGTACTCCCCAGGCGGAATGCTTAATGCGTTAGCTGCAGCACTGAAGGGCGGAAACCCTCCAACACTTAGCATTCATCGTTTACGGTATGGACTACCAGGGTATCTAATCCTGTTTGCTACCCATACTTTCGAGCCTCAGCGTCAGTTACAGACCAGACAGCCGCCTTCGCCACTGGTGTTCTTCCATATATCTACGCATTTCACCGCTACACATGGAGTTCCACTGTCCTCTTCTGCACTCAAGTTTCCCAGTTTCCGATGCACTTCTTCGGTTGAGCCGAAGGCTTTCACATCAGACTTAAAAAACCGCCTGCGCTCGCTTTACGCCCAATAAATCCGGACAACGCTTGCCACCTACGTATTACCGCGGCTGCTGGCACGTAGTTAGCCGTGGCTTTCTGGTTAAATACCGTCAATACCTGAACAGTTACTCTCAGATATGTTCTTCTTTAACAACAGAGTTTTACGAGCCGAAACCCTTCTTCACTCACGCGGCGTTGCTCCATCAGACTTTCGTCCATTGTGGAAGATTCCCTACTGCTGCCTCCCGTAGGAGTTTGGGCCGTGTCTCAGTCCCAATGTGGCCGATTACCCTCTCAGGTCGGCTACGTATCATTGCCATGGTGAGCCGTTACCCCACCATCTAGCTAATACGCCGCGGGACCATCCAAAAGTGATAGCCGAAGCCATCTTTCAAACTCGGACCATGCGGTCCAAGTTGTTATGCGGTATTAGCATCTGTTTCCAGGTGTTATCCCCCGCTTCTGGGCAGGTTTCCCACGTGTTACTCACCAGTTCGCCACTCACTCAAATGTAAATCATGATGCAAGCACCAATCAATACCAGAGTTCGTCGACTGCATGTATAG

>Lactobacillus plantarum APW1634D
GGCGGCTGGTTCCTAAAGGTTACCCCACCGACTTTGGGTGTTACAAACTCTCATGGTGTGACGGGCGGTGTGTACAAGGCCCGGGAACGTATTCACCGCGGCATGCTGATCCGCGATTACTAGCGATTCCGACTTCATGTAGGCGAGTTGCAGCCTACAATCCGAACTGAGAATGGCTTTAAGAGATTAGCTTACTCTCGCGAGTTCGCAACTCGTTGTACCATCCATTGTAGCACGTGTGTAGCCCAGGTCATAAGGGGCATGATGATTTGACGTCATCCCCACCTTCCTCCGGTTTGTCACCGGCAGTCTCACCAGAGTGCCCAACTTAATGCTGGCAACTGATAATAAGGGTTGCGCTCGTTGCGGGACTTAACCCAACATCTCACGACACGAGCTGACGACAACCATGCACCACCTGTATCCATGTCCCCGAAGGGAACGTCTAATCTCTTAGATTTGCATAGTATGTCAAGACCTGGTAAGGTTCTTCGCGTAGCTTCGAATTAAACCACATGCTCCACCGCTTGTGCGGGCCCCCGTCAATTCCTTTGAGTTTCAGCCTTGCGGCCGTACTCCCCAGGCGGAATGCTTAATGCGTTAGCTGCAGCACTGAAGGGCGGAAACCCTCCAACACTTAGCATTCATCGTTTACGGTATGGACTACCAGGGTATCTAATCCTGTTTGCTACCCATACTTTCGAGCCTCAGCGTCAGTTACAGACCAGACAGCCGCCTTCGCCACTGGTGTTCTTCCATATATCTACGCATTTCACCGCTACACATGGAGTTCCACTGTCCTCTTCTGCACTCAAGTTTCCCAGTTTCCGATGCACTTCTTCGGTTGAGCCGAAGGCTTTCACATCAGACTTAAAAAACCGCCTGCGCTCGCTTTACGCCCAATAAATCCGGACAACGCTTGCCACCTACGTATTACCGCGGCTGCTGGCACGTAGTTAGCCGTGGCTTTCTGGTTAAATACCGTCAATACCTGAACAGTTACTCTCAGATATGTTCTTCTTTAACAACAGAGTTTTACGAGCCGAAACCCTTCTTCACTCACGCGGCGTTGCTCCATCAGACTTTCGTCCATTGTGGAAGATTCCCTACTGCTGCCTCCCGTAGGAGTTTGGGCCGTGTCTCAGTCCCAATGTGGCCGATTACCCTCTCAGGTCGGCTACGTATCATTGCCATGGTGAGCCGTTACCCCACCATCTAGCTAATACGCCGCGGGACCATCCAAAAGTGATAGCCGAAGCCATCTTTCAAACTCGGACCATGCGGTCCAAGTTGTTATGCGGTATTAGCATCTGTTTCCAGGTGTTATCCCCCGCTTCTGGGCAGGTTTCCCACGTGTTACTCACCAGTTCGCCACTCACTCAAATGTAAATCATGATGCAAGCACCAATCAATACCAGAGTTCGTTCGACTTGCATGTATTAG

>Lactobacillus rhamnosus APW2288B
TTTAGACGGCTCGCTCCCTAAAGGGTTACGCCACCGGCTTCGGGTGTTACAAACTCTCATGGTGTGACGGGCGGTGTGTACAAGGCCCGGGAACGTATTCACCGCGGCGTGCTGATCCGCGATTACTAGCGATTCCGACTTCGTGTAGGCGAGTTGCAGCCTACAGTCCGAACTGAGAATGGCTTTAAGAGATTAGCTTGACCTCGCGGTCTCGCAACTCGTTGTACCATCCATTGTAGCACGTGTGTAGCCCAGGTCATAAGGGGCATGATGATTTGACGTCATCCCCACCTTCCTCCGGTTTGTCACCGGCAGTCTTACTAGAGTGCCCAACTAAATGCTGGCAACTAGTCATAAGGGTTGCGCTCGTTGCGGGACTTAACCCAACATCTCACGACACGAGCTGACGACAACCATGCACCACCTGTCATTTTGCCCCCGAAGGAGAAACCTGATCTCTCAGGTGATCAAAAGATGTCAAGACCTGGTAAGGTTCTTCGCGTTGCTTCGAATTAAACCACATGCTCCACCGCTTGTGCGGGCCCCCGTCAATTCCTTTGAGTTTCAACCTTGCGGTCGTACTCCCCAGGCGGAATGCTTAATGCGTTAGCTGCGGCACTGAAGGGCGGAAACCCTCCAACACCTAGCATTCATCGTTTACGGCATGGACTACCAGGGTATCTAATCCTGTTCGCTACCCATGCTTTCGAGCCTCAGCGTCAGTTACAGACCAGACAGCCGCCTTCGCCACTGGTGTTCTTCCATATATCTACGCATTTCACCGCTACACATGGAGTTCCACTGTCCTCTTCTGCACTCAAGTTTCCCAGTTTCCGATGCACTTCCTCGGTTAAGCCGAGGGCTTTCACATCAGACTTAAAAAACCGCCTGCGCTCGCTTTACGCCCAATAAATCCGGATAACGCTTGCCACCTACGTATTACCGCGGCTGCTGGCACGTAGTTAGCCGTGGCTTTCTGGTTGGATACCGTCACGCCGACAACAGTTACTCTGCCGACCATTCTTCTCCAACAACAGAGTTTTACGACCCGAAAGCCTTCTTCACTCACGCGGCGTTGCTCCATCAGACTTGCGTCCATTGTGGAAGATTCCCTACTGCTGCCTCCCGTAGGAGTTTGGGCCGTGTCTCAGTCCCAATGTGGCCGATCAACCTCTCAGTTCGGCTACGTATCATTGCCTTGGTGAGCCGTTACCTCACCAACTAGCTAATACGCCGCGGGTCCATCCAAAAGCGATAGCTTACGCCATCTTTCAGCCAAGAACCATGCGGTTCTTGGATTTATGCGGTATTAGCATCTGTTTCCAAATGTTATCCCCCACTTAAGGGCAGGTTACCCACGTGTTACTCACCCGTCCGCCACTCGTTCAAAATTAAATCAAGATGCAAGCACCCTTTCAATAATCAGAACTCGTTCGACTGCATGTATAG
